# Preventive Effect of *Limosilactobacillus fermentum SCHY34* on Lead Acetate-Induced Neurological Damage in SD Rats

**DOI:** 10.3389/fnut.2022.852012

**Published:** 2022-04-27

**Authors:** Xingyao Long, Haibo Wu, Yujing Zhou, Yunxiao Wan, Xuemei Kan, Jianjun Gong, Xin Zhao

**Affiliations:** ^1^Chongqing Collaborative Innovation Center for Functional Food, Chongqing Engineering Research Center of Functional Food, Chongqing Engineering Laboratory for Research and Development of Functional Food, Chongqing University of Education, Chongqing, China; ^2^Department of Neurosurgery, Chongqing Traditional Chinese Medicine Hospital, Chongqing, China

**Keywords:** *Limosilactobacillus fermentum*, lead poisoning, neurological damage, cognitive ability, oxidative stress

## Abstract

Lead poisoning caused by lead pollution seriously affects people's health. Lactic acid bacteria has been shown to be useful for biological scavenging of lead. In this experiment, Sprague-Dawley (SD) rats were treated with 200 mg/L of lead acetate solution daily to induce chronic lead poisoning, and oral *Limosilactobacillus fermentum (L. fermentum) SCHY34* to study its mitigation effects and mechanisms on rat neurotoxicity. The *L. fermentum SCHY34* showed competent results on *in vitro* survival rate and the lead ion adsorption rate. Animal experiments showed that *L. fermentum SCHY34* maintained the morphology of rat liver, kidney, and hippocampi, reduced the accumulation of lead in the blood, liver, kidney, and brain tissue. Further, *L. fermentum SCHY34* alleviated the lead-induced decline in spatial memory and response capacity of SD rats, and also regulated the secretion of neurotransmitters and related enzyme activities in the brain tissue of rats, such as glutamate (Glu), monoamine oxidase (MAO), acetylcholinesterase (AchE), cyclic adenosine monophosphate (cAMP), and adenylate cyclase (AC). In addition, the expression of genes related to cognitive capacity, antioxidation, and anti-apoptotic in rat brain tissues were increased *L. fermentum SCHY34 treatment*, such as brain-derived neurotrophic factor (BDNF), c-fos, c-jun, superoxide dismutase (SOD)1/2, Nuclear factor erythroid 2-related factor 2 (Nrf2), and B-cell lymphoma 2 (Bcl-2), and so on. *L. fermentum SCHY34* showed a great biological scavenging and potential effect on alleviating the toxicity of lead ions.

## Introduction

Lead is a multiaffinity toxic heavy metal that can accumulate in the environment over time, pollute the environment, and directly or indirectly pollute food. Lead in the environment can also enter the human body through various channels such as the respiratory tract, digestive tract, skin, and mucous membranes (1). The Joint FAO/WHO Expert Committee on Food Additives (JECFA) set the limit for daily lead intake to 1.3 μg/kg BW for adults and 0.6 μg/kg BW for children. The chemical properties of lead are relatively stable, and it does not easily decay or transfer. Lead accumulates in the body and can damage the human nervous system, reproductive system, and circulatory system, and cause damage to the corresponding tissues and organs such as the brain, kidney, liver, and cardiovascular organs (2). Children are more sensitive to the toxicity of lead than adults, and the nervous system is the most sensitive organ. Lead mainly affects the peripheral nervous system of adults and the central nervous system of children, especially the central nervous system of developing children (3). Current research shows that the direct mechanism of lead poisoning to the nervous system is mainly about: 1. Enter the brain tissue through the blood-brain barrier, combine with nerve cells in the brain tissue, change cell function and morphology, and obstruct the supply of nutrients and energy (4); 2. Cause neurotoxicity by inhibiting the release and conduction of neurotransmitters (5); 3. Competitively inhibit Ca^2+^ in the body, form a lead-calmodulin complex, affect the normal flow of Ca^2+^ in brain tissue, interfere with the uptake and release of Ca^2+^ by nerve cell membranes, disrupt intracellular Ca^2+^ homeostasis and cause neurotoxicity (6); 4. Interfere with the synthesis of brain-derived neurofactors, immediate-early genes, and synaptophysin (SYN), and affect the expression of proteins related to learning and memory, which in turn leads to impairment of learning and memory (7); 5. Inhibit the growth and repair of synapses, affecting the normal function of synapses and reducing neuronal synaptic plasticity (8).

In addition to direct damage to neurons, lead can also cause the production of oxygen free radicals in the organism (9). Free radicals cannot be excreted through metabolism and cause oxidative damage, and the organism cannot repair itself before oxidative damage occurs, leading to metabolic imbalance (3). The body contains active antioxidant substances can effectively antagonize the oxidative damage caused by the production of free radicals in the organism. Lead can reduce the activity of antioxidant enzymes in the cell, inhibit the activity of sulfhydryl-dependent enzymes, reduce the defense of the plasma membrane against reactive oxygen species (ROS), increase the lipid peroxidation of neuronal cells, and reduce active of glutathione (GSH) and superoxide dismutase (SOD). These effects induce oxidative damage to neuronal cells (10).

The traditional treatment for lead poisoning is based on the combination of metal chelating agents with vitamins. At present, the metal chelating agents commonly used in clinical treatment of lead poisoning are 2,3-dimercaptosuccinic acid (DMSA) and calcium sodium edetate (EDTA) (11). However, the efficacy of chelating agents in the treatment of lead poisoning varies greatly between individuals. Moreover, long-term use or one-time large-dose use of chelating agents can cause damage to liver and kidney function, and irreversible damage to the liver and kidneys (12). Therefore, to improve the quality of life of lead poisoning patients and reduce potential damage to groups at high risk for lead exposure, it is urgent to develop new methods for the prevention and treatment of lead poisoning.

Probiotics can colonize the host and exert probiotic effects. Several studies suggest that probiotics in humans and animals have an adsorption effect on toxic heavy metal ions (13). The possible mechanism for the detoxification of heavy metals by probiotics is that they react with heavy metals through surface adsorption, intracellular adsorption, and extracellular adsorption to purify heavy metal pollution. In addition, probiotics can also change the valence of heavy metals and reduce toxicity through oxidation or reduction (14). Lactic acid bacteria (LAB) in humans and animals are an edible probiotic that plays an important role in maintaining the microecological balance of the body and improve immune function. They have many sources, are easy to obtain, and are convenient to cultivate. In recent years, the removal of heavy metals *in vivo* and *in vitro* using LAB has become an important method for the biodegradation of heavy metals (15).

The *L. fermentum SCHY34* used in this study was isolated from the yak yogurt of Sichuan Hongyuan. *In vitro* experiments found that LF-SCHY34 exhibited effective resistance to artificial gastric acid, anti-bile salt capacity, and strong lead ion adsorption. Animal experiments verified the protective effect of *L. fermentum SCHY34* on the nerves, liver, and kidneys of lead-exposed SD rats and showed alleviation of lead-induced oxidative damage.

## Materials and Methods

### Experimental Strain

*Limosilactobacillus fermentum SCHY34* was isolated from yogurt in Hongyuan, Sichuan, China using de Man, Rogosa and Sharpe (MRS) medium. *L. fermentum SCHY34* was identified using the Basic local alignment search tool (BLAST) from the National Center of Biotechnology Information (NCBI). This strain is currently stored in the China General Microbiological Culture Collection Center (Beijing, China) and the preservation number is CGMCC No. 18795.

### Determination of Survival Rate of *L. fermentum SCHY34* in Artificial Gastric Juice

Artificial gastric juice is a mixture of 0.2% NaCl and 0.35% pepsin. The pH was adjusted to 3.0 with 1 mol/L HCl and then filtered and sterilized with a 0.22 μm sterile filter. *L. fermentum SCHY34* was activated twice in 5 ml MRS liquid medium and centrifuged at 3,000 rpm for 10 min to collect the bacteria. The bacterial pellet was washed twice with sterile saline and resuspended in 5 ml saline 1:1 (v/v), mixed with the sterile artificial gastric juice, shaken, and placed in a constant temperature incubator at 37°C. The number of viable bacteria was determined at 0 h and 3 h, and the survival rate of *L. fermentum SCHY34* in artificial gastric juice was calculated using the formula (1) (16):


(1) $$\begin{array}{l} \text{Survival rate }(\%)\;=\;\\ 3\text{ h viable count }(\text{CFU}/\text{mL})/0\text{ h viable count }(\text{CFU}/\text{mL})\\ \times 100 \end{array}$$

### Determination of the Growth Efficiency of *L. fermentum SCHY34* in Bile Salts

Activated *L. fermentum SCHY34* was inoculated twice at 2% (v/v) into sterilized MRS-THIO medium (0.2% sodium thioglycolate was added to MRS medium) containing 0.0, 0.3, and 1.0% porcine bile salts. After culturing in a constant temperature shaker at 37°C for 24 h, control blank medium (uninoculated MRS-THIO medium) and the inoculated medium were added to a 96-well plate (200 μl/well) and the Optical Density (OD) was measured at a wavelength of 600 nm. Growth efficiency was calculated using the formula (2) (17):


(2) $$\begin{array}{l} \text{Growth efficiency }(\%)=\;(\text{OD}600\text{ of }0.3\%\text{ or}1.0\%\text{bile salt medium}\\ -\text{blank medium})/(\text{OD}600\text{ of }0.0\%\text{ bile salt medium}\\ -\text{blank medium})\times 100 \end{array}$$

### *In vitro* Lead Ion Adsorption Capacity Test

*L. fermentum SCHY34* was cultured in MRS medium at 37°C for 18 h, centrifuged at 8,000 × g at 4°C for 20 min and washed twice with ultrapure water. The final concentration of *L. fermentum SCHY34* was adjusted to 1 g/L (10^7^ CFU/mL) and added 1:1 (v/v) into a 50 mg/L lead ion solution (AlCl3·6H2O). The mixture was co-cultivated at 37°C for 24 h, centrifuged at 4°C for 20 min at 8,000 × g and washed with ultrapure water twice. The supernatant was placed under an atomic absorption spectrophotometer to determine the initial lead ion concentration (*C*i) of lead ions and the post-adsorption lead ion concentration (*C*f). The adsorption capacity of *L. fermentum SCHY34* was determined using the formula (3) (18):


(3) Lead adsorption rate (%)=(Ci−Cf)/Ci× 100

### Surface Hydrophobicity Test of *L. fermentum SCHY34* Strain

The *L. fermentum SCHY34* cell concentration was adjusted with physiological saline until the OD value was 1.000 at a wavelength of 580 nm. The absorbance adjusted suspension (2 ml) was mixed with 2 ml of xylene, vortexed for 120 min, placed at room temperature for 30 min and 1 ml of the upper aqueous phase was absorbed. Normal saline was used as the blank control. The absorbance value (*A*0) of the blank control group and the sample absorbance value (*A*1) was measured at 580 nm. The surface hydrophobicity of LAB was calculated using the formula (4) (19):


(4) Surface hydrophobicity (CSH%)=(A0−A1)/A0×100

### Scanning Electron Microscope and Scanning Energy Spectrum Analysis of *L. fermentum SCHY34* Strain Before and After Adsorption of Lead Ions

Bacteria without and after absorption of a 50 mg/L lead ion solution were centrifuged at 8,000 × g for 20 min, washed with sterilized ultrapure water three times, and then poured into 1.5 mL 2.5% glutaraldehyde to fix for 1.5 h. The solution was then washed THREE times with phosphate buffer solution (PBS), centrifuged at 6,000 × g for 10 min, dehydrated once with ethanol of different concentrations (50, 70, 90, 100%), and then centrifuged at 6,000 × g for 10 min. The elute was divided with ethanol and tert-butanol mixture (v/v = 1/1) and pure tert-butanol once, centrifuged at 6,000 × g for 10 min, frozen at −20°C for 30 min, and put it into a freeze dryer for 4 h. Finally, an ion sputtering coating device was used to coat the sample with a layer of metal film at a thickness of 100–150 A. The coated sample was then put it into an observation room and the element composition was analyzed using an energy dispersive spectrometer (18).

### Transmission Electron Microscopy Analysis of *L. fermentum SCHY34* Strain Before and After the Adsorption of Lead Ions

Bacteria without the lead ion solution and bacteria after adsorption of lead ions after mixing with 50 mg/L lead ion solution were centrifuged at 8,000 × g for 20 min and fixed with 2.5% glutaraldehyde solution at 4°C overnight. Then, the fixative solution was discarded and the sample was rinsed three times with 0.1 M, pH 7.0 phosphate buffer for 15 min each time. The sample was then fixed with a 1% osmium acid solution for 1–2 h and then the osmium acid waste solution was carefully removed. The sample was rinsed three times with 0.1 M, pH 7.0 phosphate buffer for 15 min each time, and then dehydrated with ethanol solutions of different gradient concentrations (30, 50, 70, 80, 90, and 95%). Each concentration was treated for 15 min and then 100% ethanol was used for 20 min. The sample was then treated for 1 h and 3 h with a mixture of embedding agent and acetone (v/v = 1/1 and v/v = 3/1) and then treated overnight with pure embedding agent. The infiltrated sample was embedded and heated overnight at 70°C to obtain the embedded sample. After the sample was sliced, it was stained with lead citrate solution and a 50% ethanol saturated solution of uranyl acetate for 5–10 min. After drying, the sample was observed on a transmission electron microscope (18).

### Animal Experiments

**Figure 1:**
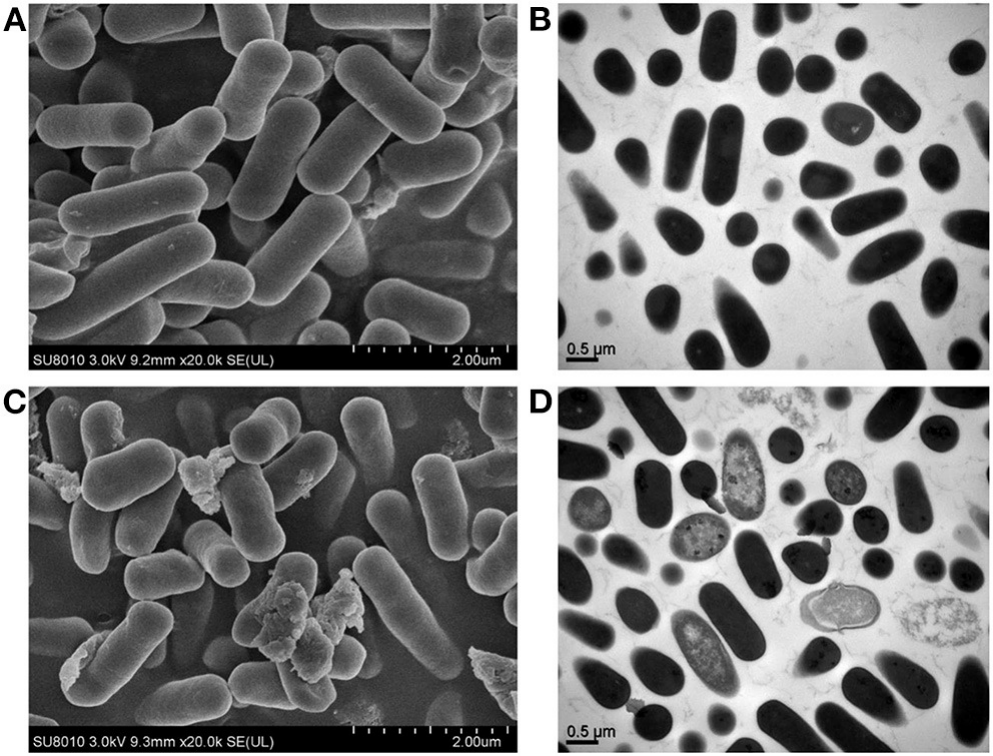
Images from scanning electron microscope (SEM) and transmission electron microscope (TEM). **(A)** SEM image of the normal group; **(B)** TEM image of blank bacterial cells; **(C)** SEM picture of lactic acid bacteria after lead adsorption; **(D)** TEM image of lead-adsorbed lactic acid bacteria cells.

After a week of adaptive feeding, 48 6-week-old SPF male SD rats were randomly divided into 4 groups: normal group (*N* = 12), lead-induced group (*N* = 12), EDTA (Sigma-Aldrich, St. Louis, MO, USA) (*N* = 12), and *L. fermentum SCHY34* group (*N* = 12). The rats in the normal group were free fed AIG-93G feed and had access to drinking water without lead acetate during the entire experimental period. The rats in the remaining three groups had access to a lead acetate solution with a concentration of 200 mg/L from the 1st week to the 12th week and had free access to AIG-93G feed. The rats in the EDTA group were injected with EDTA at a concentration of 50 mg/kg every day from the 8th week to the 12th week, and the *L. fermentum SCHY34* group was given 1 × 10^9^ CFU/kg (b.w.) *L. fermentum SCHY34* daily from the 1st week to the 12th week (Figure 1). After 12 weeks, all rats were fasted for 12 h and then anesthetized with ether. Blood was taken from the orbital vein and the mice were sacrificed. The liver, kidney, and brain tissues of the rats were collected under liquid nitrogen and stored at −80°C for later use (20).

### Morris Water Maze Experiment

The water maze had a diameter of 150 cm and a height of 50 cm and was divided into four quadrants. A platform with a height of 38 cm was set at the intersection of the four quadrants, and the water level was ~2 cm above the platform. Before daily training, the rats were placed in the water maze room to adapt for 30 min; each rat was trained once a day. The rats were placed into the water facing the wall of the pool at a fixed position in one of the four quadrants of the maze. The rats swam until they found the platform and then they were allowed to stay on the platform for 20 s. The time from when the rat went into the water until it found the platform was recorded and defined as escape latency. After 20 s of rest on the platform, the entry point was changed to another quadrant and the experiment repeated. If the platform was not found within 120 s, the rat was placed on the platform and allowed to stay for 20 s; escape latency was recorded as 120 s. On the sixth day of training, the platform was withdrawn and the water entry point remained unchanged. The rats were placed in the water for 120 s and the escape latency, residence time in the target quadrant, number of shuttles to the target location, and swimming speed were measured (ZS-001, Zhongshidichuang Technology Co., Ltd., Beijing, China) (21).

### Active Avoidance Experiment

Rats were put into any one of two chambers in the experimental box and allowed to adapt for 5 s. Then, a beeping sound lasting 20 s was initiated and a 50 V electrical stimulation given for the next 10 s. After the rats were subjected to electrical stimulation, they would move to the other chamber which was devoid of electrical stimulation. After repeated conditioning, the rats would run to the other room after receiving the conditioned stimulation (the beeping sound). This training was done 30 times each day for four consecutive days. On the fifth day, the conditioned reflex latency and the number of conditioned reflexes were tested. The mouse body tracks were recorded by Shuttle box system (22).

### Hematoxylin and Eosin and Nissl Staining, Immunohistochemical Sectioning, and Histomorphological Observation

#### H&E Staining

SD rat liver, kidney, and hippocampus tissues were fixed in 10% formalin (v/v) for 24 h. After the tissue was dehydrated, it was embedded in paraffin and then cut into 0.5 μm sections. The deparaffinized tissue was stained with hematoxylin and eosin. After dehydration, the slides were mounted with neutral gum and histological morphology was observed and photographed under an optical microscope (BX43; Olympus, Tokyo, Japan).

#### Nissl-Staining

SD rats' hippocampus tissues were fixed in 10% formalin (v/v) solution, dehydrated, and embedded in paraffin. Sections were then deparaffinized, washed with distilled water, placed in tar purple staining solution at 37°C for 10 min, and then washed with distilled water. Purple Nissl bodies were differentiated using 950 mL/L ethanol, dehydrated, cleared, and mounted. Staining was observed with a microscopic image analysis system.

#### Immunohistochemical Sectioning

The hippocampal tissue of SD rats fixed with 10% formalin (v/v) solution was dehydrated and embedded in paraffin. After sectioning, the tissue was deparaffinized and then repaired with citric acid antigen retrieval buffer at pH 6.0. A 3% hydrogen peroxide solution was used to block endogenous peroxidase, samples were blocked with serum, incubated with the primary antibody (GFAP, GB11096, Servicebio Biological Technology Co., Ltd., Wuhan, China) and then the secondary antibody (GB23303, Servicebio). Samples were then stained with diaminobenzidine (DAB, G1211, Servicebio) and counterstained with hematoxylin. Stained samples were dehydrated, mounted on slides with neutral gum, and histological morphology was observed under an optical microscope.

### Determination of Lead in Blood, Liver, Kidney, and Brain Tissues of SD Rats

Lead standard solution (0.0, 0.4, 0.8, 1.2, 1.6, 2.0 mL) was measured into a 50 mL volumetric flask and 2 mL of a mixed solution containing 12.5% ammonium dihydrogen phosphate and 2.5% magnesium nitrate was added; the volume was made up to 2 mL by 2% nitric acid. To measure the absorbance and create a standard curve, 20 μL of different concentrations of the above standard solutions were drawn into a graphite furnace atomizer.

The collected blood (500 μL) or 50 mg of each tissue was placed in a tetrafluoroethylene digestion tank and 5 mL nitric acid was added for digestion. After cooling, 1 mL of a mixed solution containing 12.5% ammonium dihydrogen phosphate and 2.5% magnesium nitrate was added, using 2% nitric acid to make the volume to 2 mL. To determine the absorbance, 20 μL of this solution was added in a Graphite furnace atomic spectrophotometer. The lead content in the blood was calculated from the standard curve Equation (5) (23).


(5) $$\begin{array}{l} \text{Lead content }(\text{μg}/\text{mL})=(9.841\times \text{ absorbance values}\\ \;-3.1831)/1000 \end{array}$$

### Determination of Oxidation Levels in Serum, Liver, Kidney, and Brain Tissues of SD Rats

Organ tissue (100 mg) was homogenized and blood samples were centrifuged to gain supernatant for the experiments. Levels of the biochemical indicators catalase (CAT), reactive oxygen species (ROS), total superoxide dismutase (T-SOD), malondialdehyde (MDA), and Glutathione (GSH) were measured according to the kit manufacturer's instructions (Nanjing Jiancheng Bioengineering Institute, China).

### Determination of Serum δ-ALAD, ALT, AST, CRE, and BUN Levels in SD Rats

Serum δ-aminolevulinic acid dehydratase (δ-ALAD), alanine aminotransferase (ALT), aspartate aminotransferase (AST), creatinine (CRE), and blood urea nitrogen (BUN) levels were measured according to the kit manufacturer's instructions (Shanghai Enzyme-linked Biotechnology Co., Ltd., Shanghai, China).

### Determination of Neurotransmitter and Related Enzyme Levels in the Brain Tissue of SD Rats

Rat brain tissue (100 mg) was homogenized with extract solution and then centrifuged to obtain the supernatant. The levels of glutamate (Glu), glutamine (Gln), glutamine synthetase (GS), monoamine oxidase (MAO), acetylcholinesterase (AchE), norepinephrine (NE), cyclic adenosine monophosphate (cAMP), and adenylate cyclase (AC) in the brain tissue were measured according to the kit manufacturer's instructions (Shanghai Enzyme-linked Biotechnology Co., Ltd., Shanghai, China).

### Analysis of mRNA in SD Rat Brain Tissue

TRIzol (Invitrogen, Carlsbad, CA, USA) was used to extract RNA from the liver tissue and the concentration was adjusted to 1 μg/μL. A cDNA reverse transcription kit (Thermo Fisher Scientific) was used to convert RNA to cDNA. The synthesized cDNA was then mixed with 10 μL SYBR Green PCR Master Mix (Thermo Fisher Scientific), 2 μL primers (Table 1), and distilled water, and then put into a qPCR instrument for processing. Quantitative PCR was performed in an automatic thermocycler for 95°C for 60 s; 40 cycles of 95°C for 15 s, 55°C for 30 s, and 72°C for 35 s; and a final step of 95°C for 30 s and 55°C for 35 s. Glyceraldehyde 3-phosphate dehydrogenase (GAPDH) was used as the internal reference gene, and the 2^−Δ*ΔCt*^ formula was used to calculate the relative mRNA transcription level.

**Table 1:** Sequences of primers.

**Gene**	**Forward sequence**	**Reverse sequence**
*BDNF*	5′-CAGCACATCCAGACAGACACCA-3′	5′-TCCAGGGCAAGCGACTCAT-3′
*c-fos*	5′-CCCACTCTGGTCTCCTCCGTG-3	5′-CTGCTCTACTTTGCCCCTTCTG-3′
*c-jun*	5′-AACGTGACCGACGAGCAGG-3	5′-ACAGCGGGAGCGACCATG-3′
*NOS1*	5′-CCTGGGGCTCAAATGGTATG-3	5′-TTGTCACAGTAGTCACGGACGC-3′
*SOD1*	5′-GCAGAAGGCAAGCGGTGAA-3	5′-GGACCGCCATGTTTCTTAGAGT-3′
*SOD2*	5′-AGCCTCCCTGACCTGCCTTAC-3	5′-CGCCTCGTGGTACTTCTCCTC-3′
*Nrf2*	5′-CAGCACATCCAGACAGACACCA-3	5′-AATATCCAGGGCAAGCGACTC-3′
*HO-1*	5′-CATGTCCCAGGATTTGTCCG-3	5′-GGGTTCTGCTTGTTTCGCTCT-3′
*Bax*	5′-GGCGATGAACTGGACAACAAC-3	5′-TAGCAAAGTAGAAAAGGGCAACC-3′
*Bcl-2*	5′-GATTGTGGCCTT CTTTGAGT-3	5′-ATAGTTCCACAAAGGCATCC-3′
*Caspase-3*	5′-AAGGAGCAGTTTTGTGTGTGTGA-3	5′-CCTGAATGATGAAGAGTTTCGG-3′
*GAPDH*	5′-AAGTTCAACGGCACAGTCAAGG-3	5′-ACGCCAGTAGACTCCACGACAT-3

### Western Blot Analysis of SD Rat Brain Tissue

Brain tissue (100 mg) was homogenized in 1 mL RIPA buffer (ThermoFisher Scientific, Waltham, MA, USA) and 10 μL PMSF (ThermoFisher Scientific) and centrifuged at 12,000 × g for 5 min at 4°C. Protein was quantified using the BCA protein assay kit (ThermoFisher Scientific). The protein sample was mixed with sample buffer (ThermoFisher Scientific) 4:1, and heated at 95°C for 5 min. The samples were then added into the wells of an SDS-PAGE gel and run at 100 V. The bands were then transferred onto PVDF membranes, the membranes were blocked with 5% skimmed milk for 1 h, and then incubated with primary antibodies (β-actin (MA5-11869); SOD1 (PA5-27240); SOD2 (PA5-30604); GSH (PA5-37307); Nuclear factor erythroid 2-related factor 2 (Nrf2) (PA5-27882); heme oxygenase 1 (HO-1) (PA5-27338); Bcl-2-associated X protein (Bax) (MA5-14003); B-cell lymphoma 2 (Bcl-2) (PA5-27094); Caspase 3 (MA1-16843); calmodulin (CaM) (PA5-82661); protein kinase A (PKA) (PA5-17626); phospho-cAMP response element-binding protein (*p*-CREB) (MA5-11192); SYN (MA5-14532) brain-derived neurotrophic factor (BDNF) (OSB00017W); N-methyl-D-aspartate receptor 1 (NMDAR1) (32–500); Nitric Oxide Synthase 1(NOS1) (61–7000); c-fos (MA5-15055); c-jun (PA5-88120) (ThermoFisher Scientific); and NMDAR2 (sc-365597, Santa Cruz, TX, USA)) overnight at 4°C. Membranes were then washed and incubated with secondary antibodies (31460; 31430, ThermoFisher Scientific) for 1 h. ECL substrate (ThermoFisher Scientific) was used to perform chemiluminescence and images were obtained using an iBright Western Blot imaging system (ThermoFisher Scientific) and analyzed.

### Statistical Aanlysis

Three measurements of serum and tissue samples were performed in parallel and the average value was calculated. SPSS software (SPSS v.25 for Windows, IBM Software Group, Chicago, IL, USA) was used to average and analyze the data. Duncan's multiple range test and One-way Analysis of Variance were used to evaluate differences between the average of each group. Differences with *p* < 0.05 were considered statistically significant.

## Results

### SD Rat *in vitro* Experimental Results

The *in vitro* antiartificial gastric juice, antibile salt experiment, surface hydrophobicity experiments, and lead ion adsorption capacity tests showed that the survival rate of *L. fermentum SCHY34* in artificial gastric juice was 88.71 ± 0.23%, the growth efficiency in 0.3 and 1.0% bile salts was 85.32 ± 0.41% and 59.31 ± 2.06%, the surface hydrophobicity rate was 43.78 ± 0.75%, and the lead ion adsorption rate was 69.58 ± 0.56%.

### SEM, Scanning Energy Spectrum, and TEM Analysis of *L. fermentum SCHY34* Bacteria Before and After the Adsorption of Lead Ions

Through observation, it was found that the normal group lactic acid bacteria cells exhibited complete morphology, clear outlines, appeared clean and plump, had a smooth surface, and no adhered particulate matter on the surface, the lead-adsorbed lactic acid bacteria had adhered into flakes and were irregularly aggregated. A large amount of adhesive material accumulated on the surface of the bacteria, and the surface of the bacteria lost its smoothness. In addition, many lactic acid bacteria cells were damaged, dented, and had collapsed (Figures 1A,C).

There were no sediments on the cut surface of the normal group of strains, the surface was clear, and there was no adhesion. Compared with the normal group of bacteria, the cut surface of the lead-adsorbed lactic acid bacterial cells exhibited a large amount of black deposits, blank areas inside the cells, the edges of the bacteria were broken, and some of the bacteria had dissolved (Figures 1B,D).

Analysis showed that after treatment with lead, the surface of the *L. fermentum SCHY34* bacteria had adsorbed a large amount of lead ions, the weight of C and P elements increased by a small amount, the weight of Pb elements increased significantly, and the weight of O and N elements decreased (Table 2; Supplementary Figure 1).

**Table 2:** Weight and atom percent before and after of adsorption of lead ions.

	**Before lead ion adsorption**		**After lead ion adsorption**	
**Element**	**Weight %**	**Atomic percentage**	**Weight %**	**Atomic percentage**
C	47.34	53.29	47.55	55.17
N	25.83	24.93	22.91	22.80
O	25.00	21.13	23.99	20.90
P	1.45	0.63	1.99	0.89
Pb	0.38	0.02	3.56	0.24
Total	100.00	100.00	100.00	100.00

### Analysis of Behavioral Indices of SD Rats

Table 3 show that in the performance of the Morris water maze experiment, the rats in the normal group had the shortest incubation period and the most shuttle times, followed by the rats in the *L. fermentum SCHY34* group, and then the rats in the EDTA group. In the active avoidance experiment, the conditioned incubation period of rats in the normal group was the shortest and the number of conditioned reflexes was the highest. The data from the rats in the *L. fermentum SCHY34* group was second only to the normal group. The incubation period of conditioned reflexes in the Morris water maze test and active avoidance experiment in the lead-induced group was significantly higher than that of the rats in other groups, and the number of shuttles and conditioned reflexes were significantly lower than that of the rats in all other groups. However, there was no significant difference in the average swimming speed of rats in all groups (Supplementary Figure 2).

**Table 3:** Rat behavioral tests.

		**Normal group**	**Lead-induced group**	**EDTA group**	***L. fermentum SCHY34* group**
Morris water maze	Incubation period (s)	20.48 ± 1.65^a^	109 ± 5.71^d^	83.65 ± 3.10^c^	52.39 ± 2.58^b^
	Number of shuttles	23.4 ± 1.54^d^	10.6 ± 1.75^a^	15.8 ± 1.15^b^	18.9 ± 1.63^c^
	Swimming speed (cm/s)	23.45 ± 1.64^a^	22.98 ± 1.01^a^	23.78 ± 0.97^a^	23.17 ± 1.33^a^
Active avoidance experiment	Conditioned reflex latency (s)	36.85 ± 7.94^a^	96.25 ± 5.41^d^	80.78 ± 6.98^c^	68.19 ± 5.76^b^
	Number of conditioned reflexes	24.16 ± 1.89^d^	12.74 ± 1.36^a^	15.15 ± 1.43^b^	20.03 ± 1.65^c^

*Value presented are the mean ± standard deviation (n = 12/group).*

a−d *Mean values with different letters in the same row are significantly different (p < 0.05) according to Duncan's multiple range test. Lead-induced group: Rat free to drink 200 mg/L lead acetate solution every day; EDTA group: Rat free to drink 200 mg/L lead acetate solution every day and treated with 500 mg/kg (b.w) EDTA every day from 8 to 12 week; L. fermentum SCHY34 group: Rat free to drink 200 mg/L lead acetate solution every day and treated with 1.0 × 10^9^ CFU/kg (b.w) of L. fermentum SCHY34 every day*.

### Histopathologic Analysis of Liver and Kidney H&E Sections, Hippocampal Nissl-Stained Sections, and Immunological Sections

**Figure 2:**
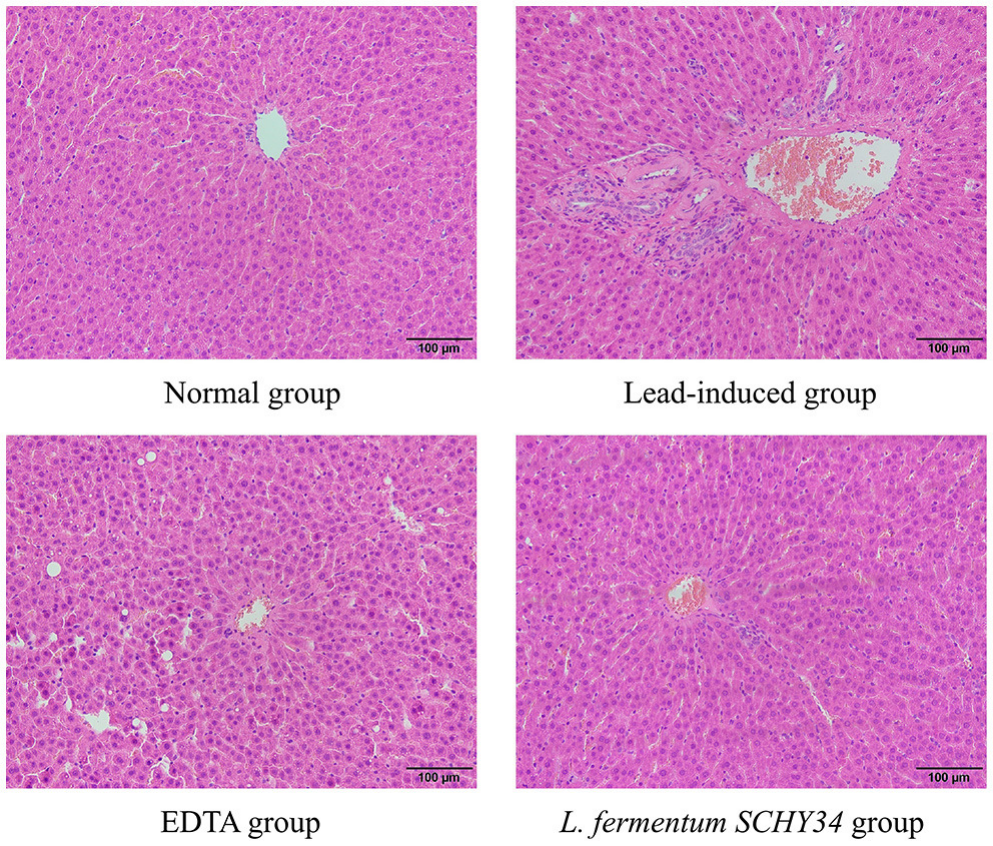
Pathological observation of liver tissue in SD rats (H&E).

In the lead-induced rats, the liver lobules were blurred, the hepatic cords disorderly, and the monocytes were scattered in different intercellular spaces after aggregation. The hepatocytes showed focal necrosis and the infiltration of large inflammatory cells, internuclear inclusions, and nuclei fragmentation. The liver cells of the rats treated with EDTA and *L. fermentum SCHY34* were more orderly, with less inflammatory cell infiltration, and less damage and necrosis of the liver cells compared with the lead-induced rats (Figure 2).

**Figure 3:**
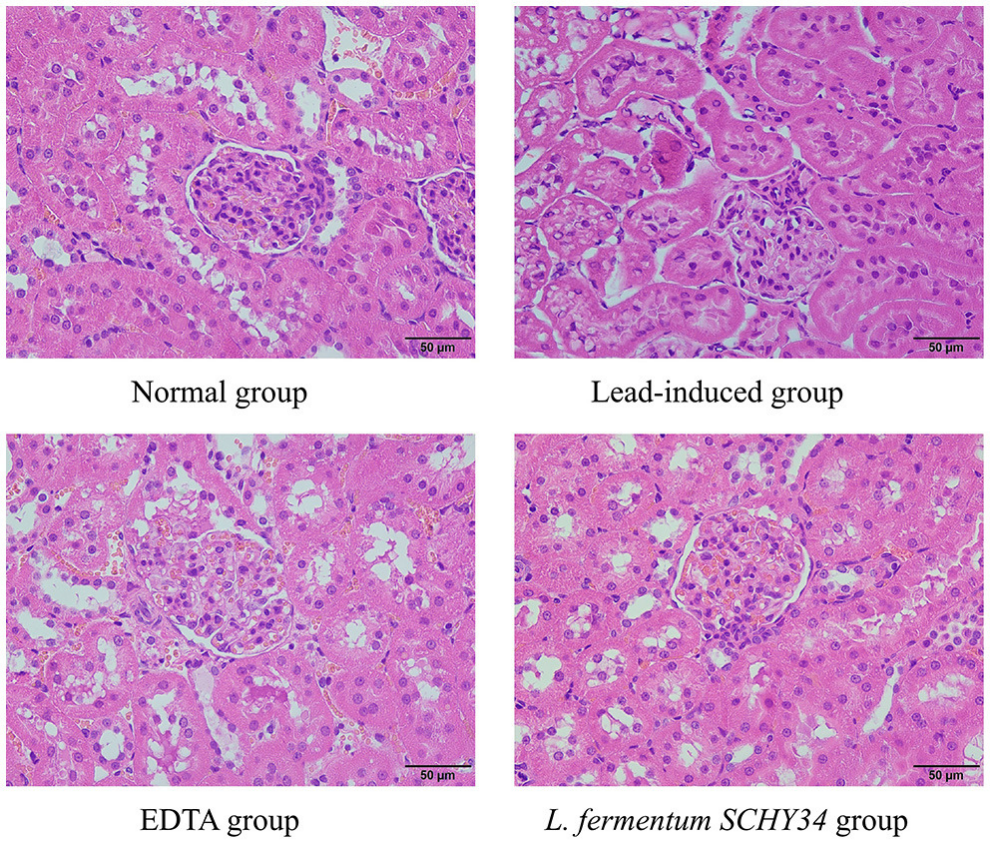
Pathological observation of kidney tissue in SD rats (H&E).

In the kidneys of SD rats in the lead-induced group, glomeruli were ruptured and the number of cell nuclei increased significantly. Glomeruli had a large number of vacuoles, the renal tubular walls were dilated with symptoms of hyperemia and swelling, epithelial cell granular degeneration, and cellular breakage, infiltrating lymphocytes, and the renal capsule cavity layer had disappeared. Compared with the lead induction group, the kidney slices of the SD rats in the EDTA and *L. fermentum SCHY34* groups had better cell integrity with less inflammatory cell infiltration and no significant renal tubular expansion. Although the structure of the glomeruli was slightly damaged, it was more complete than in the lead-induced group (Figure 3).

**Figure 4:**
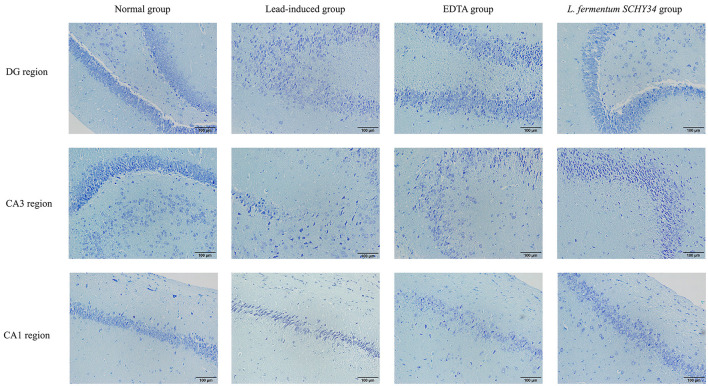
Pathological observation of hippocampus DG region, CA3 region and CA1 region in SD rats: (Nissl-stained section).

**Figure 5:**
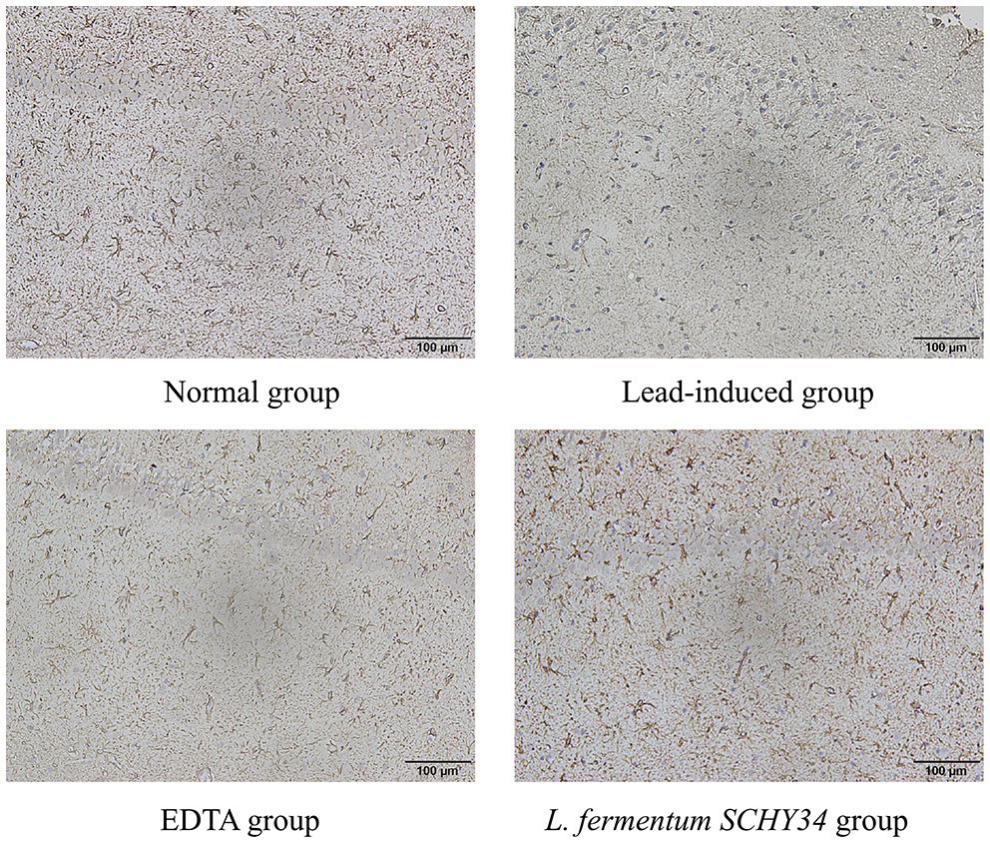
GFAP immunoreactivity of hippocampus in SD rats.

The neurons in the hippocampi of the normal group were arranged neatly and densely, the cell morphology was regular and complete, and the cytoplasm contained rich and dense Nissl bodies. The expression of astrocytes was strong, the number of cells was large, the cell body was large with dark brown-yellow staining, with thick and long protrusions, fewer branches, and a large proportion of cells. In the lead-induced rats, the CA1 area, CA3 area, and the hippocampal neurons of the dentate gyrus were scattered and loose, the neurons were missing, the shape was irregular, most were triangular or polygonal, the nucleoli were not obvious, and the Nissl bodies in the cytoplasm were reduced. The number and proportion of astrocytes in the hippocampus was significantly reduced, cell morphology was damaged, the radial neurites were shorter, and the expression level was lower. The neurons in the hippocampi of rats in the *L. fermentum SCHY34* and EDTA groups were normal in appearance and arranged neatly. The expression and cell morphology of astrocytes were similar to those in the normal group (Figures 4, 5).

The liver cell morphology, kidney cell morphology, hippocampal tissue morphology, and astrocyte morphology of SD rats treated with *L. fermentum SCHY34* were closer to those of the normal group, and the effect was better than in the EDTA group.

### Analysis of Lead Content in Blood, Liver, Kidney, and Brain Tissues of SD Rats

Results showed that the lead content in the blood, liver, kidney, and brain tissues of the normal group was the lowest among all groups. The lead content in the blood, liver, kidney, and brain tissues of rats in the lead induction group was the highest among all groups. Among these tissues, the lead content in the blood and kidneys of SD rats was the highest, followed by liver and brain tissue. The lead content in blood and tissues of the lead-induced group was approximately 15 times that of the normal group. In *L. fermentum SCHY34* group, it was 5–8 times that of the normal group, and it was 7–12 times in EDTA group (Table 4).

**Table 4:** Lead content in blood, liver, kidney, and brain tissue of SD rats.

**Group**	**Blood lead content** **(μg/L)**	**Liver lead content (μg/g)**	**Kidney lead content** **(μg/g)**	**Lead content in brain tissue** **(μg/g)**
Normal group	2.14 ± 0.30^a^	0.84 ± 0.11^a^	1.69 ± 0.18^a^	0.45 ± 0.07^a^
Lead-induced group	32.48 ± 1.28^d^	13.05 ± 0.26^d^	23.39 ± 1.97^d^	6.75 ± 0.09^d^
EDTA group	21.69 ± 0.49^c^	10.41 ± 0.20^c^	20.28 ± 0.19^c^	3.34 ± 0.06^c^
*L. fermentum SCHY34* group	18.93 ± 0.52^b^	6.16 ± 0.18^b^	13.54 ± 0.94^b^	2.48 ± 0.04^b^

*Value presented are the mean ± standard deviation (n = 12/group).*

a−d *Mean values with different letters in the same column are significantly different (p < 0.05) according to Duncan's multiple range test. Lead-induced group: Rat free to drink 200 mg/L lead acetate solution every day; EDTA group: Rat free to drink 200 mg/L lead acetate solution every day and treated with 500 mg/kg (b.w) EDTA every day from 8 to 12 week; L. fermentum SCHY34 group: Rat free to drink 200 mg/L lead acetate solution every day and treated with 1.0 × 10^9^ CFU/kg (b.w) of L. fermentum SCHY34 every day*.

### Analysis of Oxidation Levels in Serum, Liver, Kidney, and Brain Tissues of SD Rats

Through analysis of the data in Table 5, we found that the levels of CAT, T-SOD, and GSH in the blood, liver, kidney, and brain tissue of SD rats in the normal group were the highest among the four groups, and the values of MDA and ROS were the lowest. This trend was the opposite of the lead-induced rats. The blood, liver, kidney, and brain tissues of the lead-induced rats had the lowest levels of CAT, T-SOD, and GSH, while MDA and ROS were the highest among the four groups. The trend of the oxidation index results of rats in the *L. fermentum SCHY34* group was closer to that of the normal group than the rats in the EDTA group.

**Table 5:** Oxidation indexes (T-SOD, CAT, MDA, GSH, and ROS) in liver, kidney, brain tissue, and serum of SD rats.

	**Tissue/serum**	**Group**			
		**Normal group**	**Lead-induced group**	**EDTA group**	***L. fermentum SCHY34* group**
T-SOD	Liver U/mgprot	253.78 ± 14.29^d^	155.41 ± 10.76^a^	182.33 ± 9.91^b^	224.65 ± 10.37^c^
	Kidney U/mgprot	111.95 ± 4.38^d^	56.18 ± 2.56^a^	86.21 ± 4.31^b^	99.39 ± 3.68^c^
	brain tissue U/mgprot	264.72 ± 7.26^d^	94.59 ± 6.76^a^	146.34 ± 8.43^b^	200.75 ± 10.76^c^
	Serum U/mlprot	409.48 ± 10.58^d^	218.67 ± 7.29^a^	338.45 ± 15.47^b^	386.47 ± 3.85^c^
CAT	Liver U/mgprot	52.48 ± 1.71^d^	13.84 ± 0.79^a^	30.76 ± 3.01^b^	41.58 ± 1.78^c^
	Kidney U/mgprot	12.79 ± 0.64^d^	2.18 ± 0.30^a^	4.16 ± 0.71^b^	8.59 ± 0.45^c^
	brain tissue U/mgprot	45.89 ± 1.96^d^	18.15 ± 1.03^a^	28.43 ± 1.61^b^	35.97 ± 1.97^c^
	Serum U/mlprot	37.81 ± 1.45^d^	13.49 ± 1.48^a^	25.49 ± 1.56^b^	31.33 ± 1.72^c^
GSH	Liver μmol/g	416.81 ± 14.56^d^	206.81 ± 11.74^a^	290.25 ± 18.65^b^	352.24 ± 15.51^c^
	Kidney μmol/g	291.46 ± 16.45^d^	118.64 ± 15.71^a^	186.93 ± 13.52^b^	235.02 ± 15.23^c^
	brain tissue μmol/g	354.32 ± 10.53^d^	178.52 ± 17.10^a^	221.85 ± 18.79^b^	295.11 ± 10.25^c^
	Serum μmol/l	286.57 ± 15.22^d^	104.82 ± 14.67^a^	195.64 ± 13.71^b^	247.50 ± 11.63^c^
MDA	Liver nmol/mgprot	1.11 ± 0.04^a^	3.35 ± 0.20^d^	2.87 ± 0.14^c^	1.58 ± 0.19^b^
	Kidney nmol/mgprot	0.57 ± 0.14^a^	3.30 ± 0.54^d^	2.16 ± 0.21^c^	1.40 ± 0.17^b^
	brain tissue nmol/mgprot	7.49 ± 0.45^a^	28.56 ± 0.12^d^	18.43 ± 0.25^c^	12.71 ± 0.57^b^
	Serum nmol/mlprot	1.45 ± 0.09^a^	7.97 ± 0.53^d^	5.36 ± 0.41^c^	3.04 ± 0.30^b^
ROS	Liver (×10^4^)	2.32 ± 0.19^a^	6.41 ± 0.12^d^	3.61 ± 0.17^c^	2.88 ± 0.13^b^
	Kidney (×10^4^)	1.94 ± 0.41^a^	5.82 ± 0.21^d^	6.57 ± 0.23^c^	4.99 ± 0.17^b^
	brain tissue (×10^4^)	0.94 ± 0.14^a^	6.14 ± 0.32^d^	3.87 ± 0.16^c^	2.60 ± 0.37^b^
	Serum (×10^4^)	2.74 ± 0.10^a^	9.96 ± 0.39^d^	6.68 ± 0.29^c^	4.56 ± 0.26^b^

*Value presented are the mean ± standard deviation (n = 12/group).*

a−d *Mean values with different letters in the same row are significantly different (p < 0.05) according to Duncan's multiple range test. Lead-induced group: Rat free to drink 200 mg/L lead acetate solution every day; EDTA group: Rat free to drink 200 mg/L lead acetate solution every day and treated with 500 mg/kg (b.w) EDTA every day from 8 to 12 week; L. fermentum SCHY34 group: Rat free to drink 200 mg/L lead acetate solution every day and treated with 1.0 ×10^9^ CFU/kg (b.w) of L. fermentum SCHY34 every day*.

### Analysis of Serum ALT, AST, BUN, CRE, and δ-ALAD Indices in SD Rats

Among the four groups of rats, the normal group had the lowest ATL and AST enzyme activities, the highest δ-ALAD enzyme activities, and the BUN and CRE content was the lowest. Due to the effect of lead ions, the activity of ATL, AST, and the content of BUN and CRE were the highest, and the δ-ALAD enzyme activity was the lowest in the lead-induced group. The ALT and AST enzyme activities and the content of BUN and CRE in the EDTA and *L. fermentum SCHY34* groups were significantly lower than in the lead induction group. In addition, the δ-ALAD enzyme activity was significantly higher than that of the lead induction group and the intervention effect of *L. fermentum SCHY34* was better than EDTA (Table 6).

**Table 6:** ALT, AST, BUN, CRE, and δ-ALAD in the serum of SD rats.

**Groups**	**ALT** **(μmol/L)**	**AST** **(μmol/L)**	**BUN** **(μmol/L)**	**CRE** **(μmol/L)**	**δ-ALAD** **(μmol/L)**
Normal group	31.06 ± 2.52^a^	58.51 ± 2.78^a^	1344.85 ± 48.76^a^	29.48 ± 1.21^a^	505.04 ± 16.98^d^
Lead-induced group	63.79 ± 2.42^d^	87.43 ± 2.85^d^	2070.89 ± 49.60^d^	42.12 ± 1.14^d^	351.47 ± 15.74^a^
EDTA group	51.46 ± 2.87^c^	76.67 ± 2.45^c^	1859.42 ± 36.81^c^	34.49 ± 1.82^c^	390.24 ± 17.85^b^
*L. fermentum SCHY34* group	42.30 ± 1.65^b^	69.65 ± 2.74^b^	1609.09 ± 37.64^b^	31.12 ± 1.34^b^	435.74 ± 15.87^c^

*Value presented are the mean ± standard deviation (n = 12/group).*

a−d *Mean values with different letters in the same column are significantly different (p < 0.05) according to Duncan's multiple range test. Lead-induced group: Rat free to drink 200 mg/L lead acetate solution every day; EDTA group: Rat free to drink 200 mg/L lead acetate solution every day and treated with 500 mg/kg (b.w) EDTA every day from 8 to 12 week; L. fermentum SCHY34 group: Rat free to drink 200 mg/L lead acetate solution every day and treated with 1.0 ×10^9^ CFU/kg (b.w) of L. fermentum SCHY34 every day*.

### Analysis of Neurosignaling Substance Levels in the Brain Tissue of SD Rats

The brain tissue of SD rats in the normal group had the lowest glutamate content and the lowest monoamine oxidase activity. The other neurotransmitters and enzymes had the highest content and the highest activity in all groups. After lead exposure, the lead-induced rats had the highest Glu content and the highest MAO activity. The content and activities of Gln, NE, cAMP, GS, AchE, and AC were the lowest of the four groups. Both *L. fermentum SCHY34* and EDTA effectively alleviated the changes of lead ions induced on neurotransmitters and enzymes in brain tissue; *L. fermentum SCHY34* had a better alleviating effect than EDTA (Table 7).

**Table 7:** Glu, Gln, GS, MAO, AchE, NE, cAMP, AC in the brain tissue of SD rats.

	**Normal group**	**Lead-induced group**	**EDTA group**	***L. fermentum SCHY34* group**
Glu (μmol/gprot)	49.38 ± 0.59^a^	139.41 ± 1.68^d^	93.31 ± 1.01^c^	65.66 ± 0.85^b^
Gln (μmol/gprot)	331.58 ± 9.48^d^	104.76 ± 5.17^a^	194.33 ± 7.53^b^	260.85 ± 7.69^c^
GS (U/gprot)	103.94 ± 5.90^d^	46.78 ± 2.59^a^	55.67 ± 2.41^b^	76.38 ± 3.08^c^
MAO (U/gprot)	38.14 ± 6.87^a^	193.63 ± 4.83^d^	128.67 ± 3.39^c^	75.92 ± 6.51^b^
AchE (U/gprot)	143.18 ± 3.62^d^	55.74 ± 1.43^a^	79.06 ± 2.79^b^	117.54 ± 4.78^c^
NE (pg/mL)	376.91 ± 11.35^d^	190.46 ± 8.30^a^	250.73 ± 8.85^b^	309.82 ± 13.79^c^
cAMP (μmol/gprot)	238.14 ± 6.87^d^	128.67 ± 3.39^a^	145.92 ± 6.51^b^	193.63 ± 4.83^c^
AC (U/gprot)	77.68 ± 2.36^d^	23.15 ± 0.79^a^	43.56 ± 1.12^b^	59.62 ± 1.75^c^

*Value presented are the mean ± standard deviation (n = 12/group).*

a−d *Mean values with different letters in the same row are significantly different (p < 0.05) according to Duncan's multiple range test. Lead-induced group: Rat free to drink 200 mg/L lead acetate solution every day; EDTA group: Rat free to drink 200 mg/L lead acetate solution every day and treated with 500 mg/kg (b.w) EDTA every day from 8 to 12 week; L. fermentum SCHY34 group: Rat free to drink 200 mg/L lead acetate solution every day and treated with 1.0 ×10^9^ CFU/kg (b.w) of L. fermentum SCHY34 every day*.

### Analysis of mRNA and Protein Expression in SD Rat Brains

**Figure 6:**
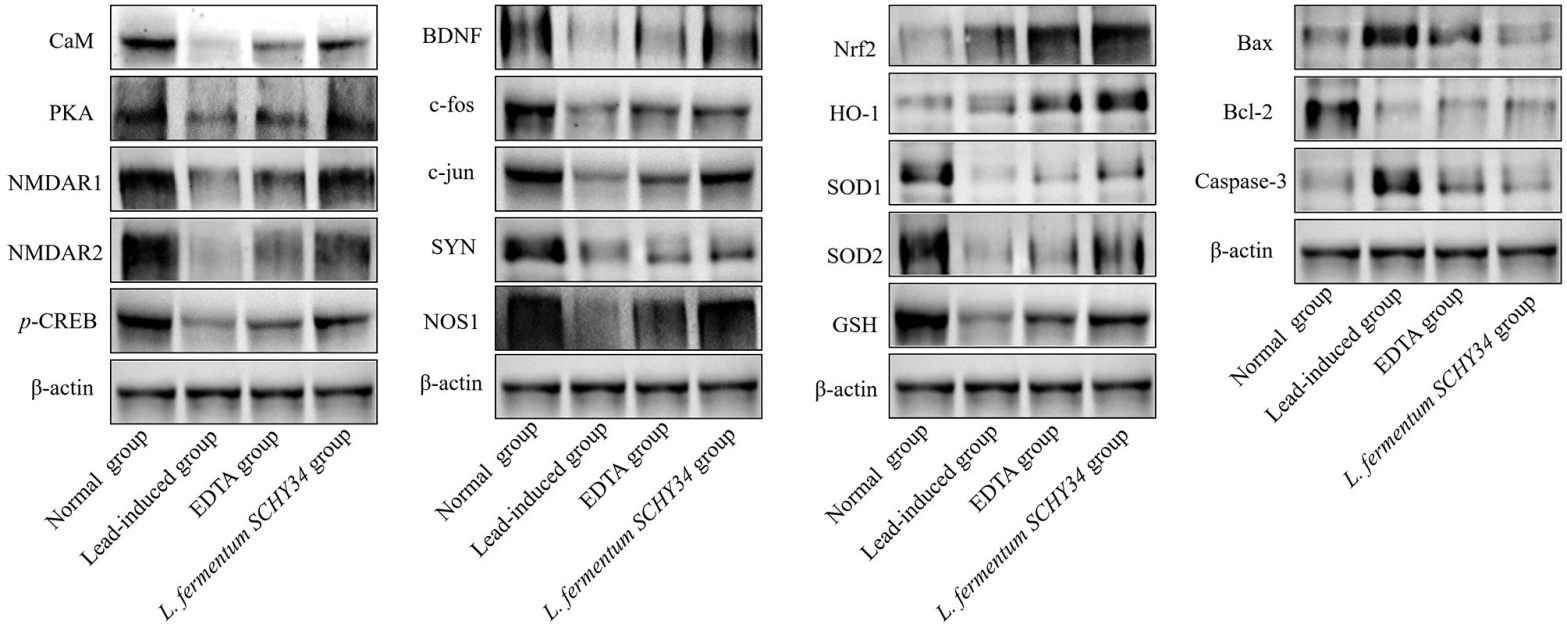
Protein expression in brain tissue of SD rats.

In the brain tissues of normal SD rats, the mRNA and protein expression of BDNF and the early genes c-fos and c-jun was the highest. In addition, the expression of oxidation related SOD1, SOD2, and NOS1 was the highest, the expression of Nrf2 and HO-1 was the lowest, the expression of apoptosis related Bax and Caspase-3 was the lowest, and the expression of Bcl-2 was the highest. The expression of the BDNF, c-fos, c-jun, SOD1, SOD2, NOS1, and Bcl-2 genes in the brain tissue of the lead-induced group was the lowest and Bax and Caspase-3 were the highest. In addition, the expression of Nrf2 and HO-1 was slightly higher than that of the normal group, but much lower than the mRNA expression in the brain tissue of rats in the *L. fermentum SCHY34* and EDTA groups (Table 8; Figure 6).

**Table 8:** Brain tissue mRNA expression in SD rats.

	**BDNF**	**c-fos**	**c-jun**	**Nrf2**	**HO-1**	**SOD1**	**SOD2**	**Bax**	**Bcl-2**	**Caspase-3**
Normal group	9.15 ± 0.46^d^	7.69 ± 0.18^d^	6.91 ± 0.40^d^	0.87 ± 0.25^a^	0.94 ± 0.33^a^	10.24 ± 1.04^d^	8.86 ± 0.42^d^	0.25 ± 0.04^a^	6.38 ± 0.24^d^	0.17 ± 0.05^a^
Lead-induced group	1.00 ± 0.00^a^	1.00 ± 0.00^a^	1.00 ± 0.00^a^	1.00 ± 0.00^a^	1.00 ± 0.00^a^	1.00 ± 0.00^a^	1.00 ± 0.00^a^	1.00 ± 0.00^d^	1.00 ± 0.00^a^	1.00 ± 0.00^d^
EDTA group	3.66 ± 0.51^b^	2.11 ± 0.16^b^	2.97 ± 0.34^b^	4.76 ± 0.38^b^	3.89 ± 0.57^b^	5.56 ± 0.42^b^	2.44 ± 0.34^b^	0.78 ± 0.16^c^	2.73 ± 0.14^b^	0.71 ± 0.03^c^
*L. fermentum SCHY34* group	6.03 ± 0.27^c^	5.18 ± 0.19^c^	5.17 ± 0.23^c^	7.58 ± 0.94^c^	6.83 ± 0.45^c^	8.68 ± 0.51^c^	5.61 ± 0.37^c^	0.57 ± 0.07^b^	4.68 ± 0.21^c^	0.29 ± 0.04^b^

*Value presented are the mean ± standard deviation (n = 12/group).*

a−d *Mean values with different letters in the same column are significantly different (p < 0.05) according to Duncan's multiple range test. Lead-induced group: Rat free to drink 200 mg/L lead acetate solution every day; EDTA group: Rat free to drink 200 mg/L lead acetate solution every day and treated with 500 mg/kg (b.w) EDTA every day from 8 to 12 week; L. fermentum SCHY34 group: Rat free to drink 200 mg/L lead acetate solution every day and treated with 1.0 ×10^9^ CFU/kg (b.w) of L. fermentum SCHY34 every day*.

The protein expression of CaM, PKA, NMDAR1, NMDAR2, SYN, GSH, and *p*-CREB had the strongest protein expression in the brain tissue of rats in the normal group, and their expression in the brain tissue of rats in the lead-induced group was the lowest. The *L. fermentum SCHY34* group had the second highest protein expression intensity, followed by the EDTA group (Figure 6).

## Discussion

Due to environmental pollution, pollution of food and daily necessities, household pollution, poor hygiene, and eating habits, hyperleademia and lead poisoning have become modern diseases in developed and developing countries (24). Lead exerts strong neurotoxicity and is particularly harmful to brain development and the nervous system (25). As an edible probiotic, lactic acid bacteria create a healthy intestinal environment for the host by regulating the balance of bacterial populations and secreting beneficial metabolites (26). In recent years, studies have been carried out using lactic acid bacteria to ameliorate lead poisoning. After entering the body, the lactic acid bacteria first pass through the mouth, esophagus, and stomach, and then enter the intestine and begin to function. Therefore, tolerance to gastric juice and intestinal juice determines the number of lactic acid bacteria that pass through the oral cavity and enter the intestine (27). The stronger the tolerance, the greater the number of viable bacteria that can survive. The hydrophobicity of the surface of lactic acid bacteria reflects the adhesion ability of lactic acid bacteria. The stronger the hydrophobicity, the more effectively the probiotics can interact with the intestinal epithelial cells (28). The survival rate of *L. fermentum SCHY34* in artificial gastric juice was 84.3 ± 3.1% and the surface hydrophobicity was 43.8 ± 0.7%, which is significantly higher than the survival rate of lactobacillus TC50 in gastric juice is 70% and the surface hydrophobicity rate is 28.94 ± 7.5% in report by Soundharrajan et al. (29). The growth efficiency in 0.3 and 1.0% bile salts was 85.3 ± 0.4% and 59.31 ± 2.06%, which is lower than 79% of *L. plantarum* C11 with 1.0% bile salt (17). The possible reason is we treated the *L. fermentum SCHY34* in 1.0% bile salt for 24 h, but *L. plantarum* C11 treated only 4 h. The results demonstrate that *L. fermentum SCHY34* is able to pass more through the digestive system and colonize in human intestine.

The adsorption of Pb^2+^ by bacterial strains is mainly due to functional groups such as -OH, -NH, and -COOH participating in the adsorption process (26). The mechanism of adsorption includes mainly surface electrostatic interaction, complexation, ion exchange, and intracellular accumulation. In addition, macromolecular substances such as nucleic acids, phosphate esters, polysaccharides, S-layer proteins, and fatty acids also participate in the adsorption process (30). From the experimental results, after lead adsorption, *L. fermentum SCHY34* bacteria showed a large amount of aggregation under the electron microscope. The elements O, N decreased, and the elements C, P, Pb increased, indicating that the -NH and -COOH on the cell surface of *L. fermentum SCHY34* bacteria participated in the adsorption of lead-hydroxyapatite. *L. fermentum SCHY34* bacteria removed 69.6 ± 0.6% of the lead ions in the solution *in vitro*, which is much higher than the 25% lead ion removal capacity of *Lactobacillus* reported by Monachese (13). It decreased the lead in rat blood and tissue with a high efficiency as Xiao's results (31). This also indicates that *L. fermentum SCHY34* has a very strong lead adsorption capacity.

Lead in the human body is mainly excreted through the kidneys. When the maximum excretion of lead by the kidneys is reached, lead is deposited in the proximal tubule epithelial cells, affecting cell metabolism and damaging the structure and function of the kidneys. When cells are damaged or necrotic, renal tubular reabsorption function decreases, causing creatinine (CRE) and urea to remain in the blood, which resulting in an increase in blood creatinine and blood urea nitrogen concentration (32). Lead also inhibits the activity of δ-aminolevulinic acid dehydratase (δ-ALAD), which increases the ALA in the blood. δ-ALA is excreted in the urine, resulting in a decrease in the δ-ALA content in the blood. The liver is the most important detoxification organ (33). Experiments have shown that lead can cause different degrees of liver disease, cause severe inflammation, affect the activity of liver-related enzymes, and ultimately cause liver damage. ALT and AST are distributed in liver cells. When liver cells are damaged, ALT and AST in the cytoplasm is released into the blood. Therefore, lead will result in elevated AST and ALT levels in the serum (34). Through the detection of serum indices, *L. fermentum SCHY34* can increase the δ-ALAD, decrease the ALT, AST, CRE, and BUN. As well as the pathological analysis of liver and kidney sections, we found that *L. fermentum SCHY34* bacteria protected the integrity of liver and kidney cells, and relieved the liver and kidney damage in SD rats induced by lead, the same as Zhai et al. (35) and Muhammad et al. (36).

The process of learning to escape from a water environment reflects the learning ability of the animal. Spatial positioning according to the surrounding environment and purposefully swimming to a safe place in the water (platform) reflects the animal's spatial memory capacity (37). The active avoidance experiment can reflect the reaction ability and memory capacity of the rat (38). It can be seen from the rat behavioral test, *L. fermentum SCHY34* group had a short incubation period, rapid active avoidance, and better memory.

The hippocampus is an important part of the brain responsible for learning and memory (39). In the hippocampus, the DG area plays a vital role in the separation of patterns, or distinguishing similar field patterns, similar events, or similar spatial locations. The CA3 area is involved in memory recovery or pattern completion, i.e., responding to incomplete stimuli by recalling previously stored information. The CA1 area plays an important role in short-term learning and spatial patterns of objects and events. The CA1 and CA3 areas of the hippocampus are rich in location cells. Therefore, the CA1 and CA3 areas also play an important role in spatial navigation (40). The rat brain tissue slices showed that *L. fermentum SCHY34* bacteria maintained the morphology and number of nerve cells in the DG, CA1, and CA3 regions of the rat hippocampus.

Astrocytes perform many functions in the brain and are a bridge between the peripheral environment and the central nervous system. Astrocytes not only participate in the composition of the blood-brain barrier, but also maintain the stability of the internal environment of the nervous system. They also participate in the elimination of metabolites produced by neuronal activities, such as glutamate and potassium ions, and secrete cytokines to mediate the immune response of the nervous system. Astrocytes can also release neurotransmitters, participate in the transmission and integration of nerve signals, adjust neuron excitability and synaptic conduction efficiency, affect the formation of synapses and the regulation of synaptic plasticity, and play an important role in learning and memory (41, 42). GFAP-positive astrocytes can be arranged regularly in the form of an obvious lamellar structure in the hippocampus. This orderliness is conducive to establishing a fixed positional relationship and a stable functional relationship between neurons, to better regulate the functional activity of neurons (43). The *L. fermentum SCHY34* bacteria protected astrocytes from lead toxicity, and stabilized the hippocampal structure. Combining the behavioral test, these results indicate that *L. fermentum SCHY34* bacteria ameliorated the damage caused by lead to the learning and memory capacity of the rats, and protected the normal function of rat brain tissue, the same as Alves et al. (44).

Astrocytes in the brain can take up most of the glutamate in the intercellular space through glutamate transporters and generate glutamine under the catalysis of glutamine synthetase. Glutamine is then released from astrocytes and taken back into neurons, where it is hydrolyzed into glutamate. Some is converted to γ-aminobutyric acid and the rest is transported to synaptic vesicles to participate in a new round of excitement responses (45). MAO mainly exists on the surface of the mitochondrial membranes of cells in the central nervous system and can degrade NE and other monoamine neurotransmitters (46). NE is a very important class of catecholamines that is widely distributed in the central nervous system. NE can project to multiple brain regions, including the hippocampus, amygdala, and striatum. It plays a vital role in wakefulness, attention, reward, learning and memory functions, learning and memory related to stress, and synaptic plasticity (47). When MAO activity in the central nervous system increases, the catabolism of monoamine neurotransmitters such as NE increases, and symptoms such as memory loss and depression may occur (48). The activity of MAO is also an important factor affecting the generation of free radicals. Increased MAO will promote the generation of free radicals. Excess free radicals produce toxic effects, attack mitochondrial membranes, and further damage nerve cells (49). AchE is a key enzyme in biological nerve conduction. It can degrade acetylcholine, block the excitatory effect of neurotransmitters on the postsynaptic membrane, and ensure the normal transmission of nerve signals in the organism. AchE is also involved in the development and maturation of nerve cells and can promote neuronal development and nerve regeneration (50).

cAMP is an important substance involved in the regulation of substance metabolism and biological functions in cells. It is the “second messenger” of information transmission and participates in the process of learning and memory (51). It is currently believed that when certain nerve cells are excited, the presynaptic nerve terminals release transmitters to act on the corresponding receptors on the postsynaptic membrane to activate AC and catalyze the synthesis of adenosine triphosphate (ATP) in the postsynaptic membrane, which in turn activates PKA (52). PKA activation causes phosphorylation of the downstream target cyclic adenosine *p*-CREB (53). *p*-CREB promotes the transcription of BDNF, immediate early genes c-fos and c-jun, and SYN, and forms new synaptic connections. It also promotes the expression of the antiapoptotic protein gene Bcl-2 to promote the survival of nerve cells and increase synaptic plasticity (54). BDNF plays an important role in synapse remodeling in the process of animal learning, memory, and cognition. Combined with its specific receptor tyrosine kinase receptor B (TrkB), it induces phosphorylation at specific sites of the TrkB receptor and transmits BDNF signals to the nucleus for neuroprotection (55). The immediate-early genes c-fos and c-jun belong to a class of proto-oncogenes which can be induced by second messengers to respond quickly to external stimuli such as neurotransmitters, hormones, and nerve impulses (56). These genes express their expression products as third messengers to participate in the regulation of the transduction of signals closely related to learning and memory in cells (57). After normal learning and memory activities or after learning and memory impairment, regular changes in their expression occur (58). SYN is a membrane protein closely related to the structure and function of synapses. It forms synaptic vesicle-specific membrane channels, participates in the transport and discharge of vesicles, and can also be used as a presynaptic terminal specific marker (59). NMDA is an effector receptor of ionotropic glutamate, which plays an important role in synaptic excitatory conduction, synaptic plasticity, learning, and excitotoxicity. Glutamate binds to the NMDA receptor, causing the Ca^2+^ channel to open. After Ca^2+^ enters the cell, it activates CaM, which further activates nitric oxide synthase (nNOS) and AC (60). nNOS produces nitric oxide in the nervous tissues of the central nervous system and peripheral nervous system and assists in cell communication and association with native membranes (61).

When lead ions enter the brain tissue, it damages astrocytes, inhibits the activity of glutamine synthetase in the cerebral cortex, prevents glutamate from synthesizing glutamine, and causes excess glutamate to accumulate in the astrocytes of the cerebral cortex (62). The excess glutamate counteracts the glutamate/aspartate transporter (GLAST) and glutamate transporter-1 (GLT-1) distributed on the cell membrane to reduce the reuptake of glutamate, thereby causing the accumulation of glutamate in the contact gap, leading to a series of symptoms of central nervous system excitement, ultimately leading to nervous system damage (63). In addition, lead can also activate the activity of MAO, produce ROS, and cause oxidative damage in brain tissues (64). In addition, lead inhibits the activities of GS and AchE, reduces the secretion of NE, and damages the normal activities of brain tissue (65). Lead also competitively binds to related proteins to inhibit Ca^2+^ influx and disrupt intracellular Ca^2+^ balance, thereby inhibiting CaM activation and the secretion of nNOS. This leads to inhibition of AC and cAMP, affecting PKA activation and CREB phosphorylation, ultimately suppressing the expression of BDNF, C-fos, c-jun, and SYN. *L. fermentum SCHY34* bacteria can alleviate the neurotoxicity of lead to the brain tissue of SD rats, maintain the normal secretion and activity of various neurotransmitters and related enzymes, such as increase Gln, GS, AchE, NE, cAMP, AC, decrease Glu, MAO, and ensure that the Ca^2+^ channel is unblocked, thereby ensuring the supply of brain neurotrophic factors and energy, the same as Shaban's results (66). *L. fermentum SCHY34* bacteria can also activate the Nrf2/HO-1 antioxidant pathway and increase the expression of downstream SOD1, SOD2, and GSH, thereby reducing oxidative damage of brain tissue caused by lead. In addition, *L. fermentum SCHY34* bacteria can inhibit the expression of the apoptosis-related genes Bax and Caspase-3, and increase the expression of the anti-apoptotic gene Bcl-2, promoting the survival of nerve cells, similar results with Shao et al. (67).

## Conclusions

*L. fermentum SCHY34* exhibits high antiacid and antibile salt capacity, high hydrophobicity and significant lead ion adsorption ability *in vitro*. *L. fermentum SCHY34* can prevent lead ions from entering the blood-brain barrier and protecting the integrity of brain tissue cells and tissues. It can also regulate the release of neurotransmitters and related enzymes, promote the expression of cAMP and downstream related genes, activate the antioxidant pathway Nrf2/HO-1 and the expression of the anti-apoptotic gene Bcl-2, maintain the normal function of synapses and the normal activities of brain tissue. In summary, *L. fermentum SCHY34* has a strong protective effect on the structure and functional activities of brain tissue exposed to lead ions. This provides new ideas for multiple bio-utilization methods of lactic acid bacteria.

## Data Availability Statement

The raw data supporting the conclusions of this article will be made available by the authors, without undue reservation.

## Ethics Statement

The protocol for these experiments was approved by the Ethics Committee of Chongqing Collaborative Innovation Center for Functional Food (202006023B), Chongqing, China. The experimental process was in accordance with 2010/63/EU directive.

## Author Contributions

XL and HW are mainly responsible for the content of the experiment and manuscript writing. YZ, YW, XK, and JG are mainly involved in data analysis research. XZ oversaw the research and reviewed the final manuscript. All authors contributed to the article and approved the submitted version.

## Funding

This research was funded by Chongqing University Innovation Research Group Project (CXQTP20033), the Science and Technology Project of Chongqing (cstc2021jcyj-msxmX0408), and Scientific and Technological Innovation Project of Construction of Double City Economic Circle in Chengdu-Chongqing Area of Chongqing Education Commission (KJCX2020052), China.

## Conflict of Interest

The authors declare that the research was conducted in the absence of any commercial or financial relationships that could be construed as a potential conflict of interest.

## Publisher's Note

All claims expressed in this article are solely those of the authors and do not necessarily represent those of their affiliated organizations, or those of the publisher, the editors and the reviewers. Any product that may be evaluated in this article, or claim that may be made by its manufacturer, is not guaranteed or endorsed by the publisher.
