# Supplementary figures and images for: Preventive Effect of Limosilactobacillus fermentum SCHY34 on Lead Acetate-Induced Neurological Damage in SD Rats
Source: Front Nutr. 2022 Apr 27;9:852012. doi: 10.3389/fnut.2022.852012 (PMC9094495; doi:10.3389/fnut.2022.852012)

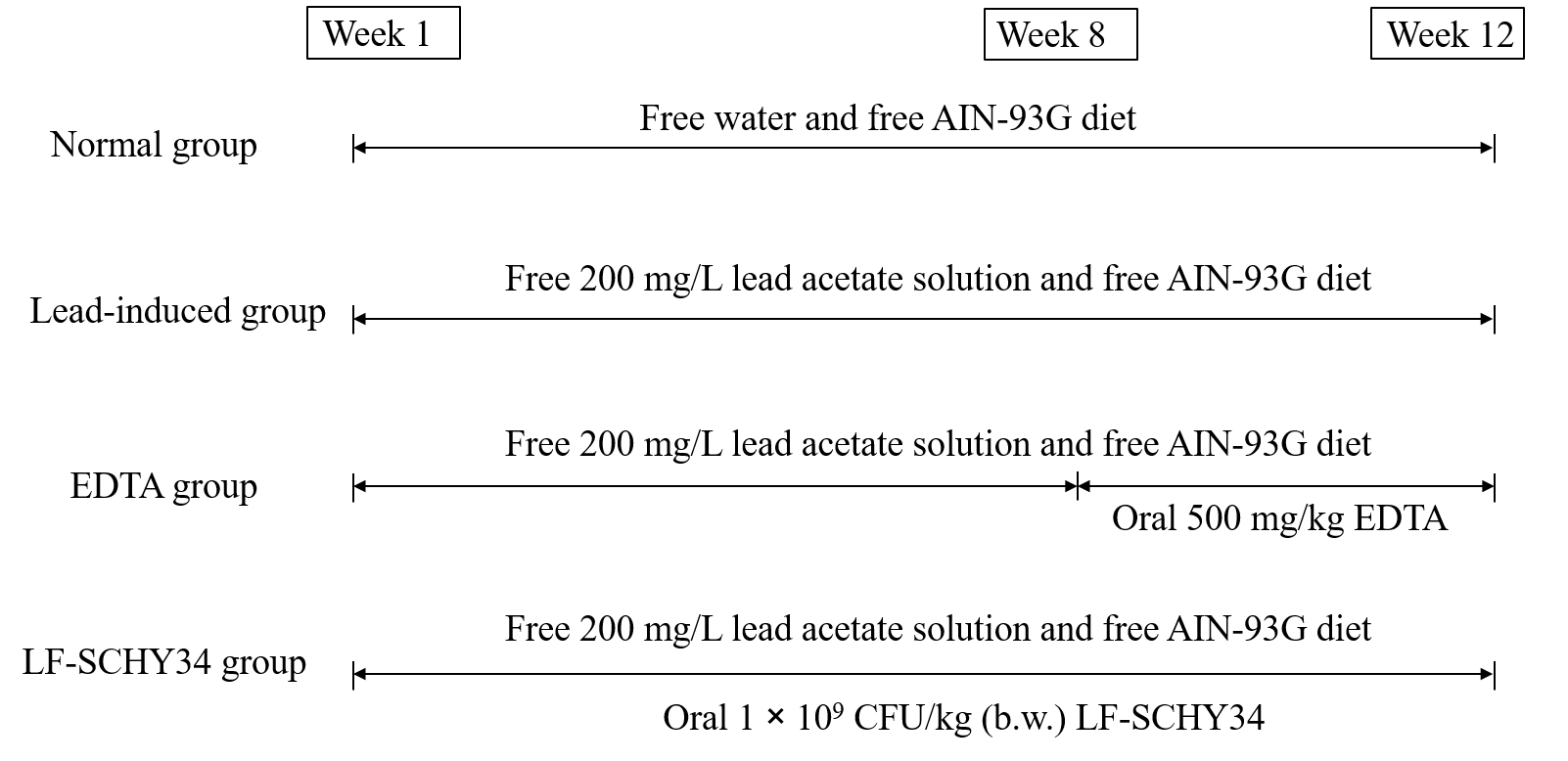

Supplement: Supplementary file 1 [file Data_Sheet_1.ZIP › SCHY34-raw data/figure 1.png]

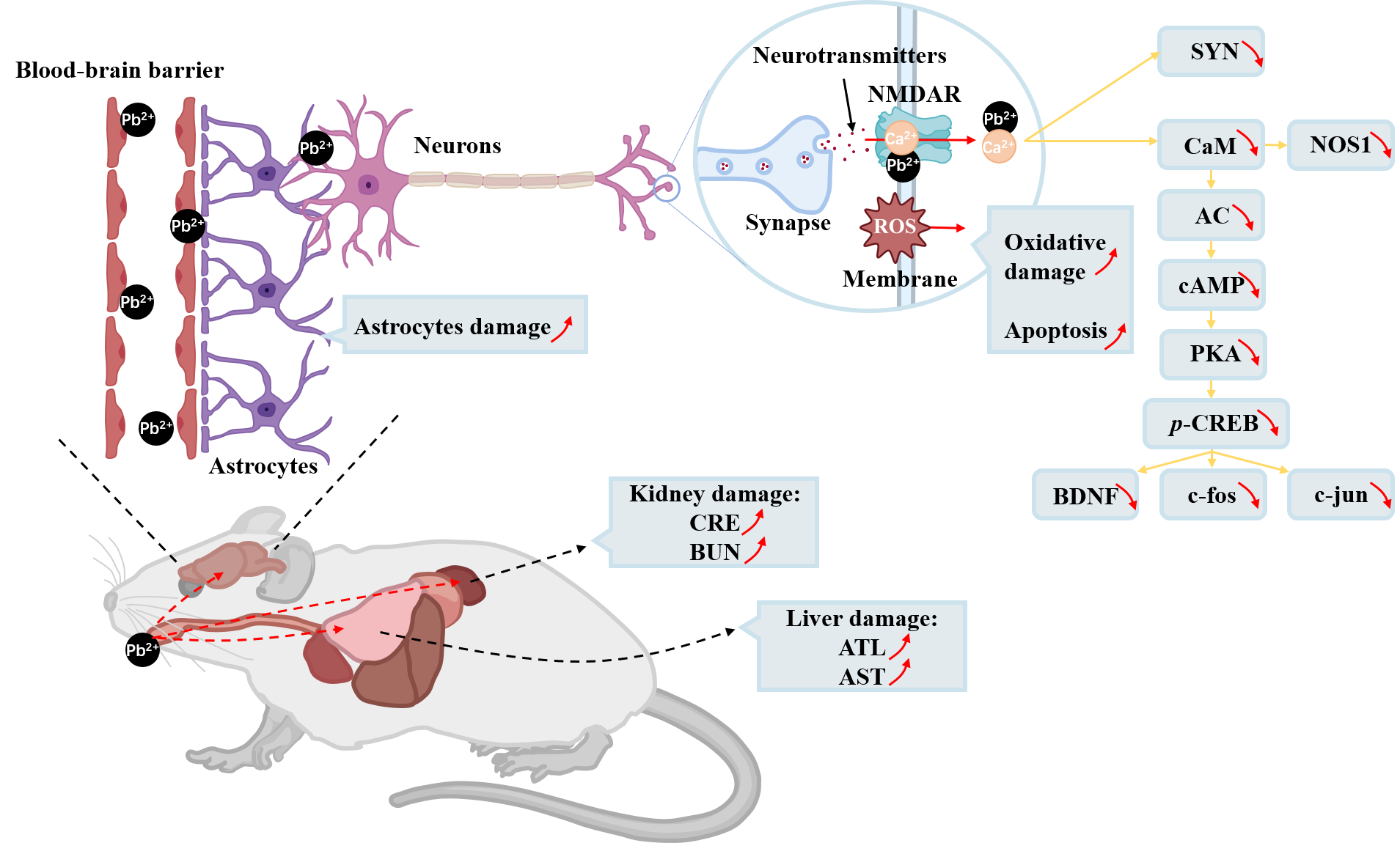

Supplement: Supplementary file 1 [file Data_Sheet_1.ZIP › SCHY34-raw data/figure 10.png]

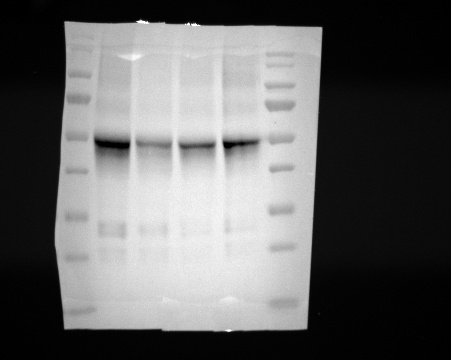

Supplement: Supplementary file 1 [file Data_Sheet_1.ZIP › SCHY34-raw data/figure 9/p-CREB.jpg]

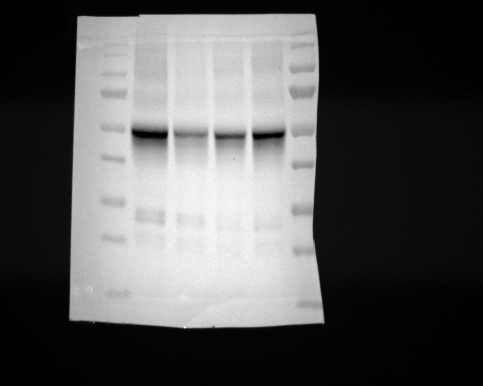

Supplement: Supplementary file 1 [file Data_Sheet_1.ZIP › SCHY34-raw data/figure 9/c-jun.jpg]

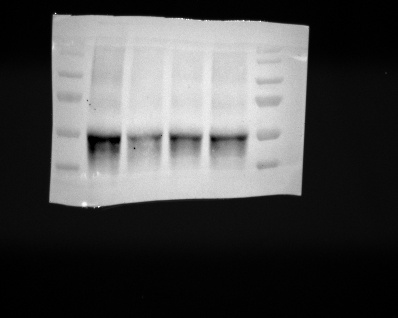

Supplement: Supplementary file 1 [file Data_Sheet_1.ZIP › SCHY34-raw data/figure 9/c-FOS.jpg]

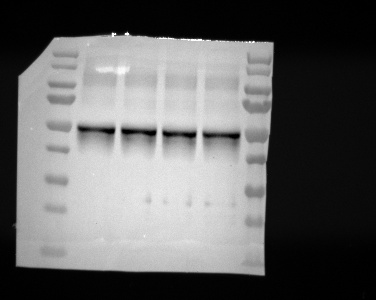

Supplement: Supplementary file 1 [file Data_Sheet_1.ZIP › SCHY34-raw data/figure 9/beta-actin.jpg]

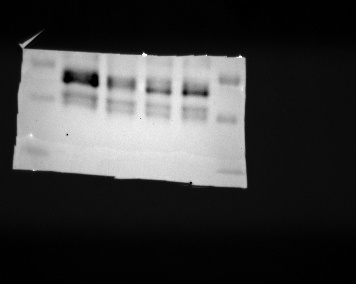

Supplement: Supplementary file 1 [file Data_Sheet_1.ZIP › SCHY34-raw data/figure 9/SYN.jpg]

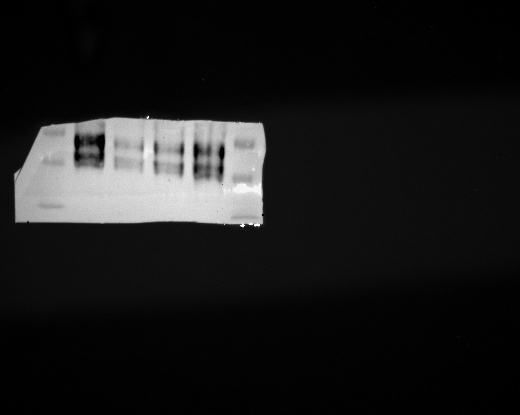

Supplement: Supplementary file 1 [file Data_Sheet_1.ZIP › SCHY34-raw data/figure 9/SOD2.jpg]

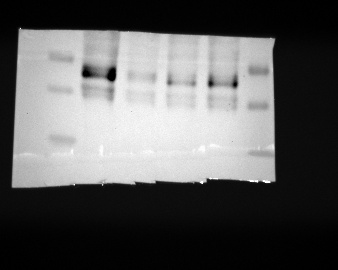

Supplement: Supplementary file 1 [file Data_Sheet_1.ZIP › SCHY34-raw data/figure 9/SOD1.jpg]

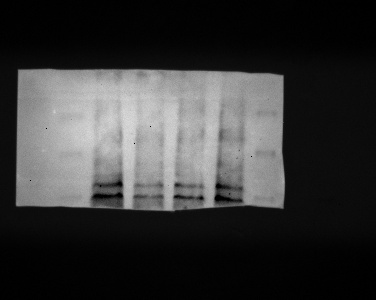

Supplement: Supplementary file 1 [file Data_Sheet_1.ZIP › SCHY34-raw data/figure 9/PKA.jpg]

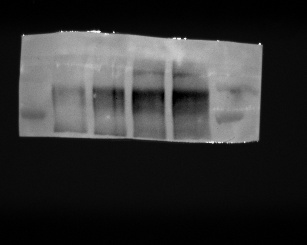

Supplement: Supplementary file 1 [file Data_Sheet_1.ZIP › SCHY34-raw data/figure 9/Nrf2.jpg]

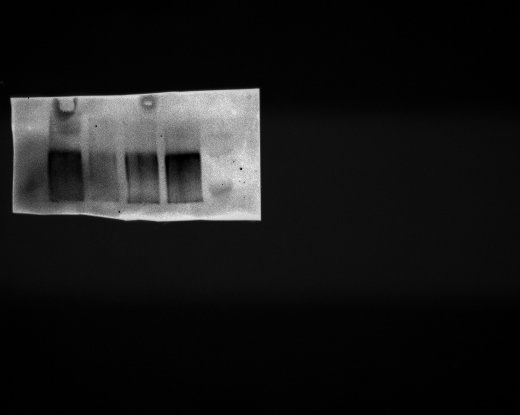

Supplement: Supplementary file 1 [file Data_Sheet_1.ZIP › SCHY34-raw data/figure 9/NOS1.jpg]

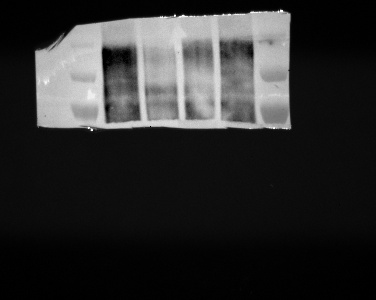

Supplement: Supplementary file 1 [file Data_Sheet_1.ZIP › SCHY34-raw data/figure 9/NMDAR2.jpg]

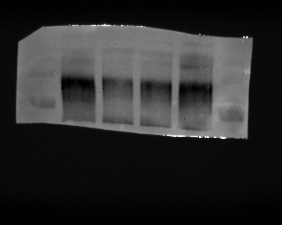

Supplement: Supplementary file 1 [file Data_Sheet_1.ZIP › SCHY34-raw data/figure 9/NMDAR1.jpg]

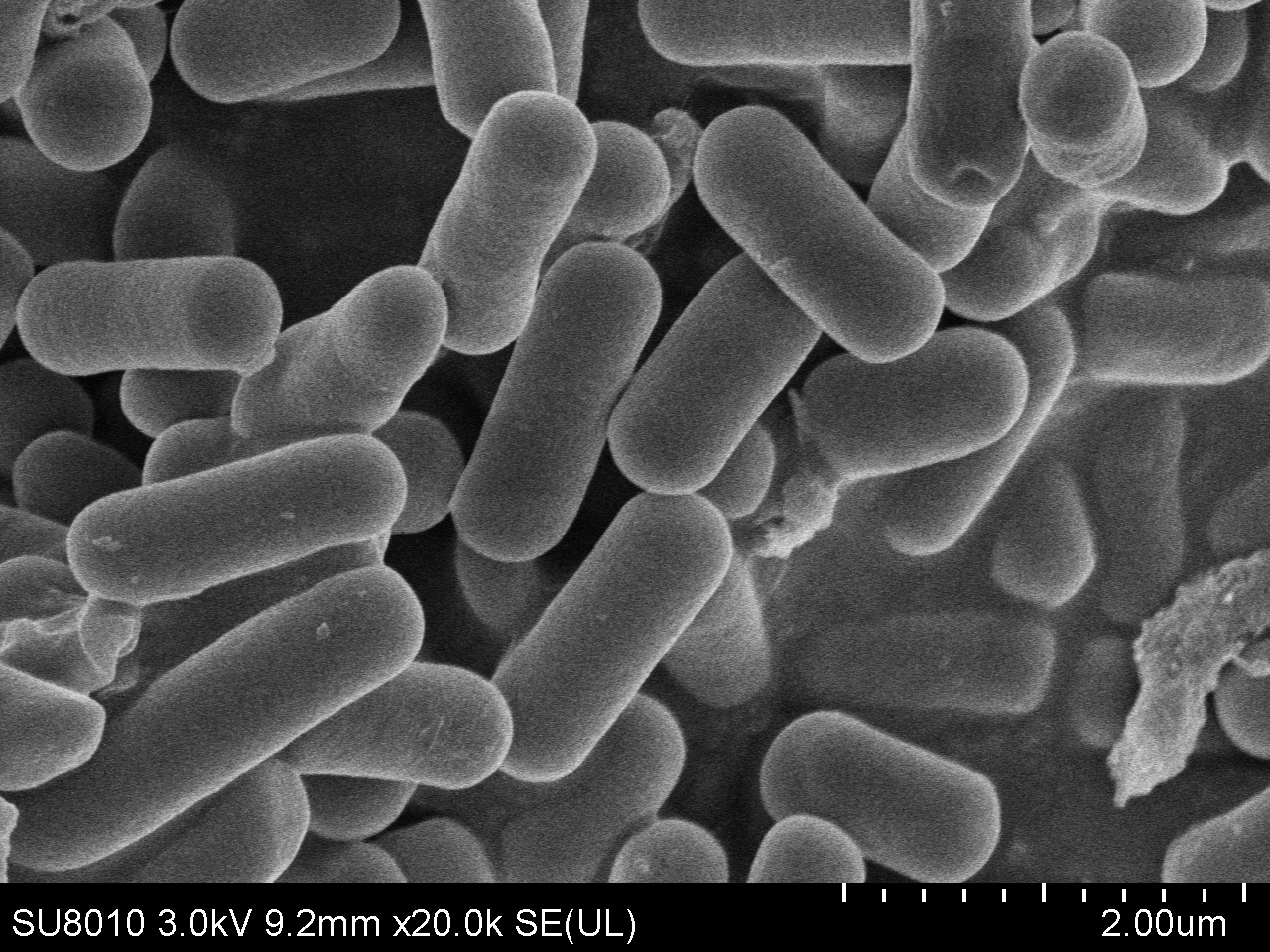

Supplement: Supplementary file 1 [file Data_Sheet_1.ZIP › SCHY34-raw data/figure 2/a.tif]

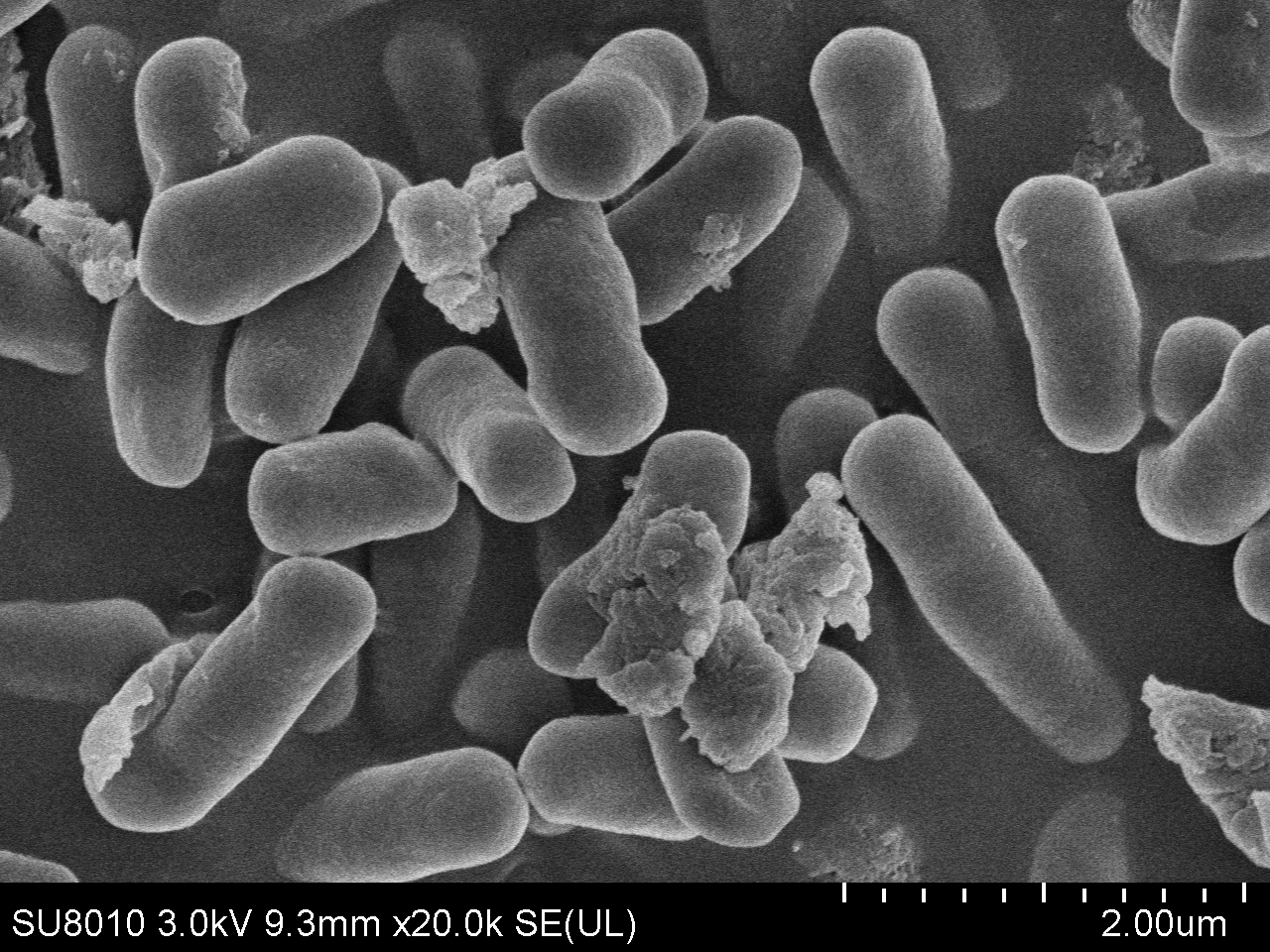

Supplement: Supplementary file 1 [file Data_Sheet_1.ZIP › SCHY34-raw data/figure 2/c.tif]

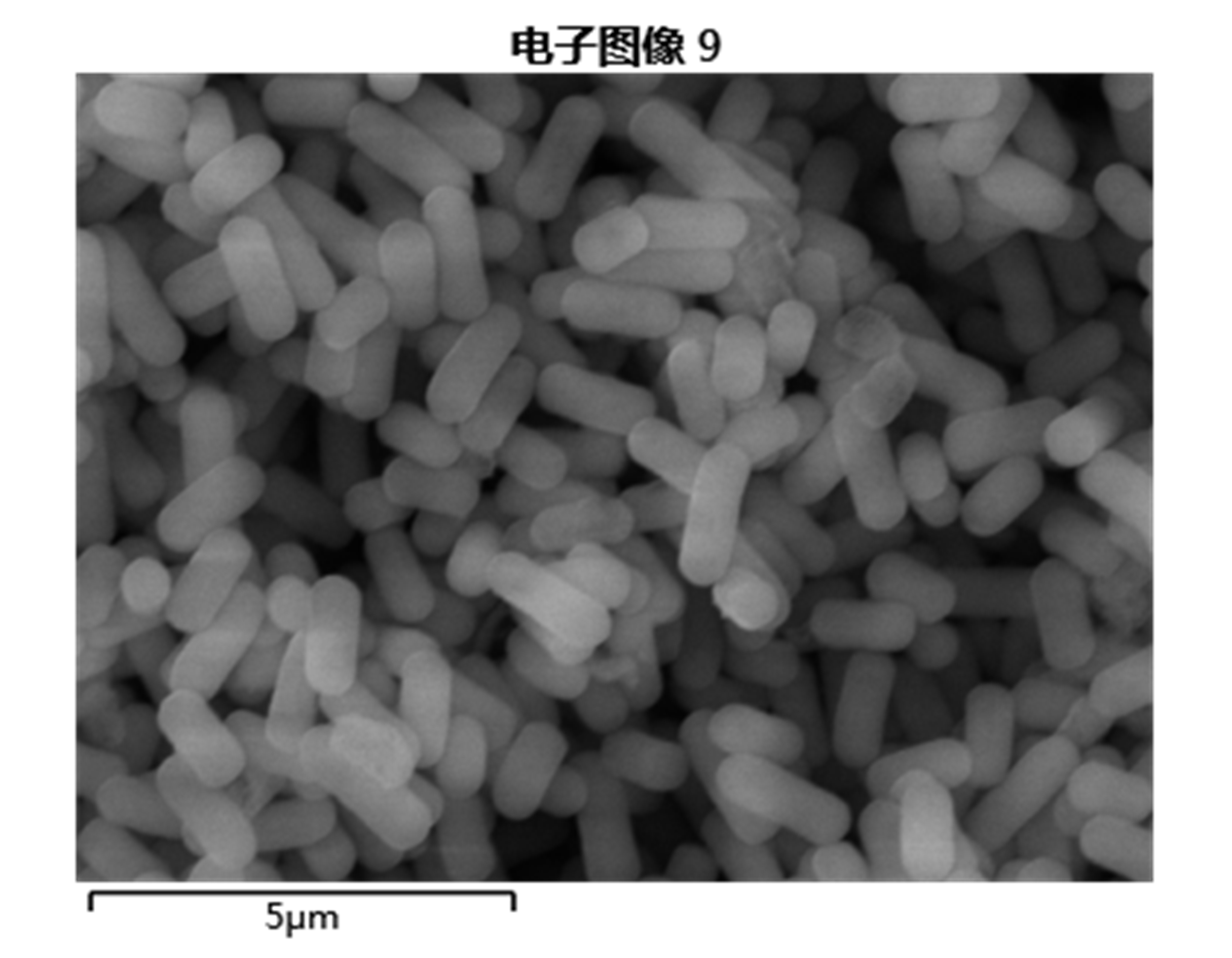

Supplement: Supplementary file 1 [file Data_Sheet_1.ZIP › SCHY34-raw data/figure 3/a.jpg]

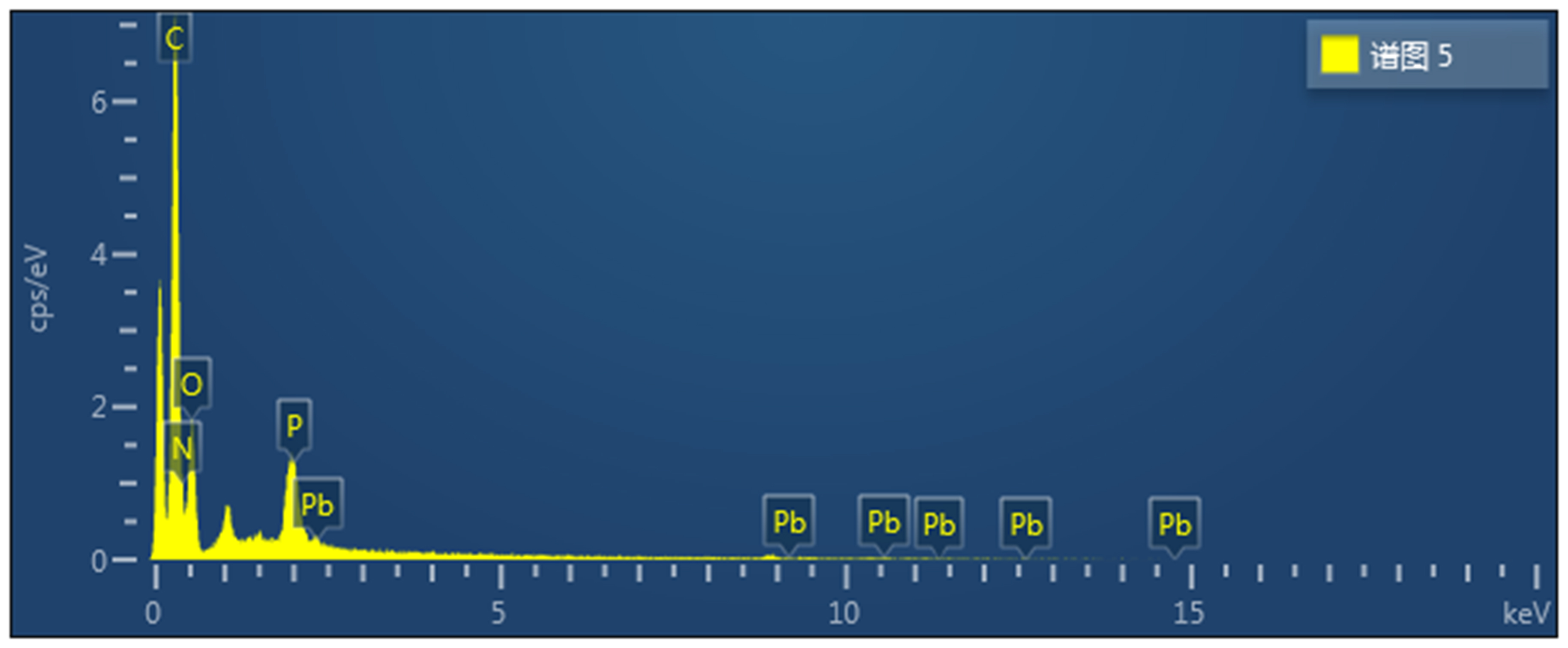

Supplement: Supplementary file 1 [file Data_Sheet_1.ZIP › SCHY34-raw data/figure 3/b.jpg]

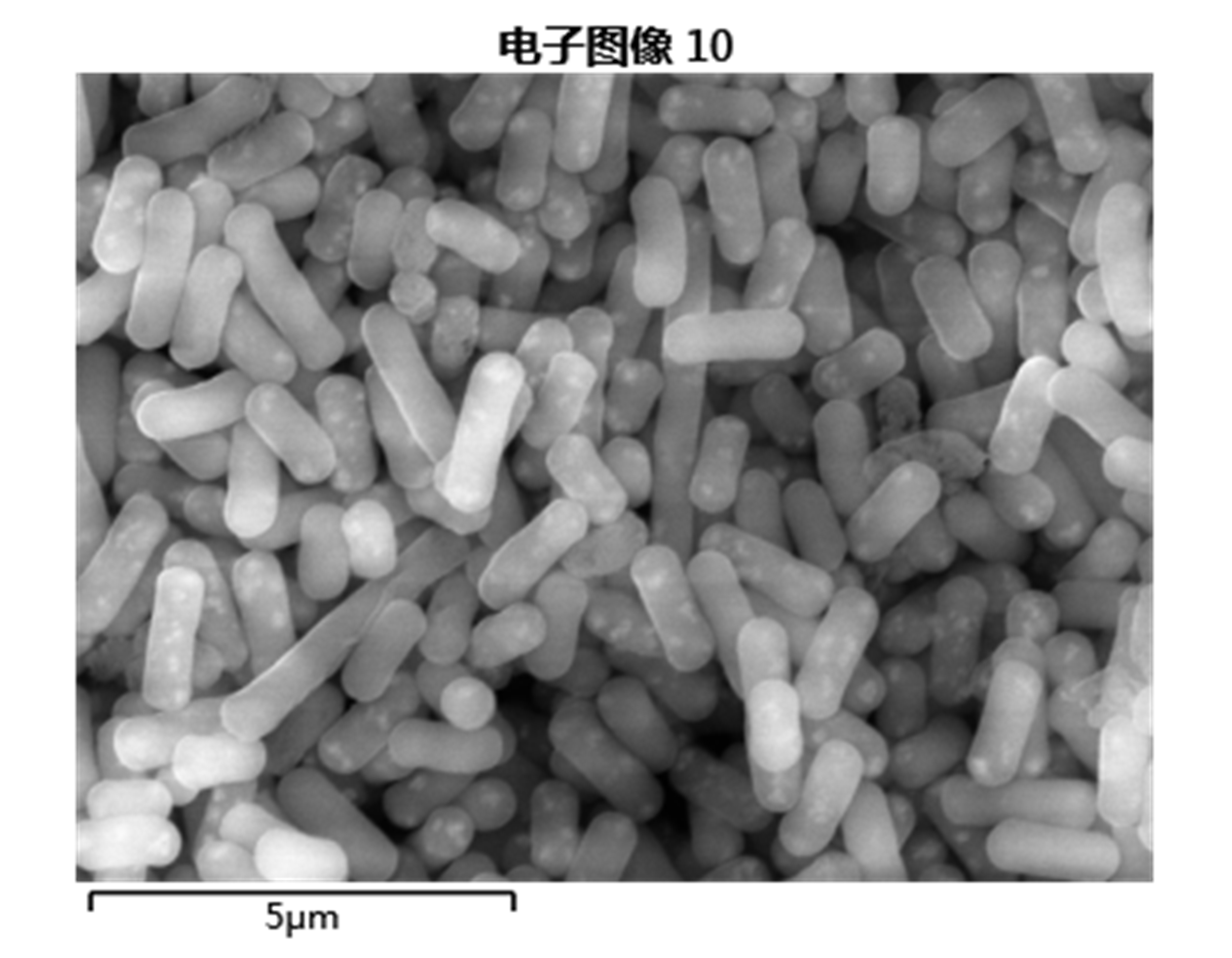

Supplement: Supplementary file 1 [file Data_Sheet_1.ZIP › SCHY34-raw data/figure 3/c.jpg]

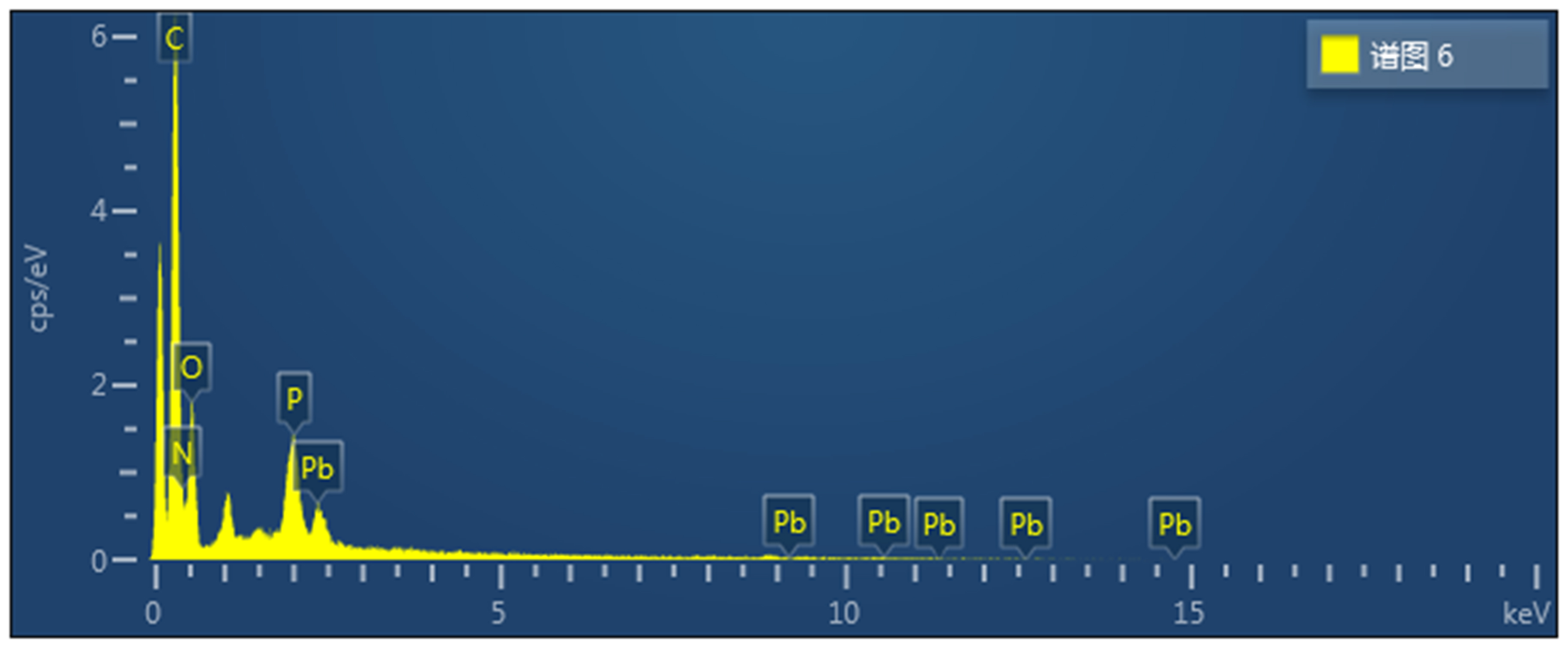

Supplement: Supplementary file 1 [file Data_Sheet_1.ZIP › SCHY34-raw data/figure 3/d.jpg]

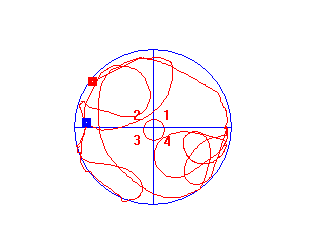

Supplement: Supplementary file 1 [file Data_Sheet_1.ZIP › SCHY34-raw data/figure 4/EDTA.bmp]

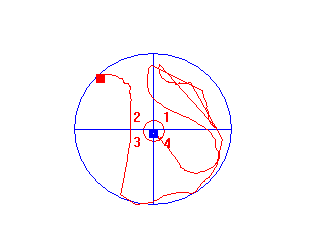

Supplement: Supplementary file 1 [file Data_Sheet_1.ZIP › SCHY34-raw data/figure 4/LF-SCHY34.bmp]

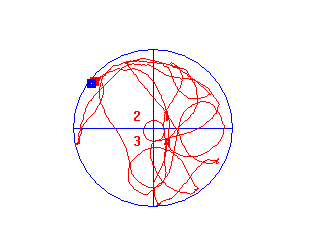

Supplement: Supplementary file 1 [file Data_Sheet_1.ZIP › SCHY34-raw data/figure 4/lead-induced.bmp]

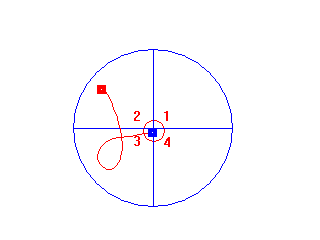

Supplement: Supplementary file 1 [file Data_Sheet_1.ZIP › SCHY34-raw data/figure 4/normal.bmp]

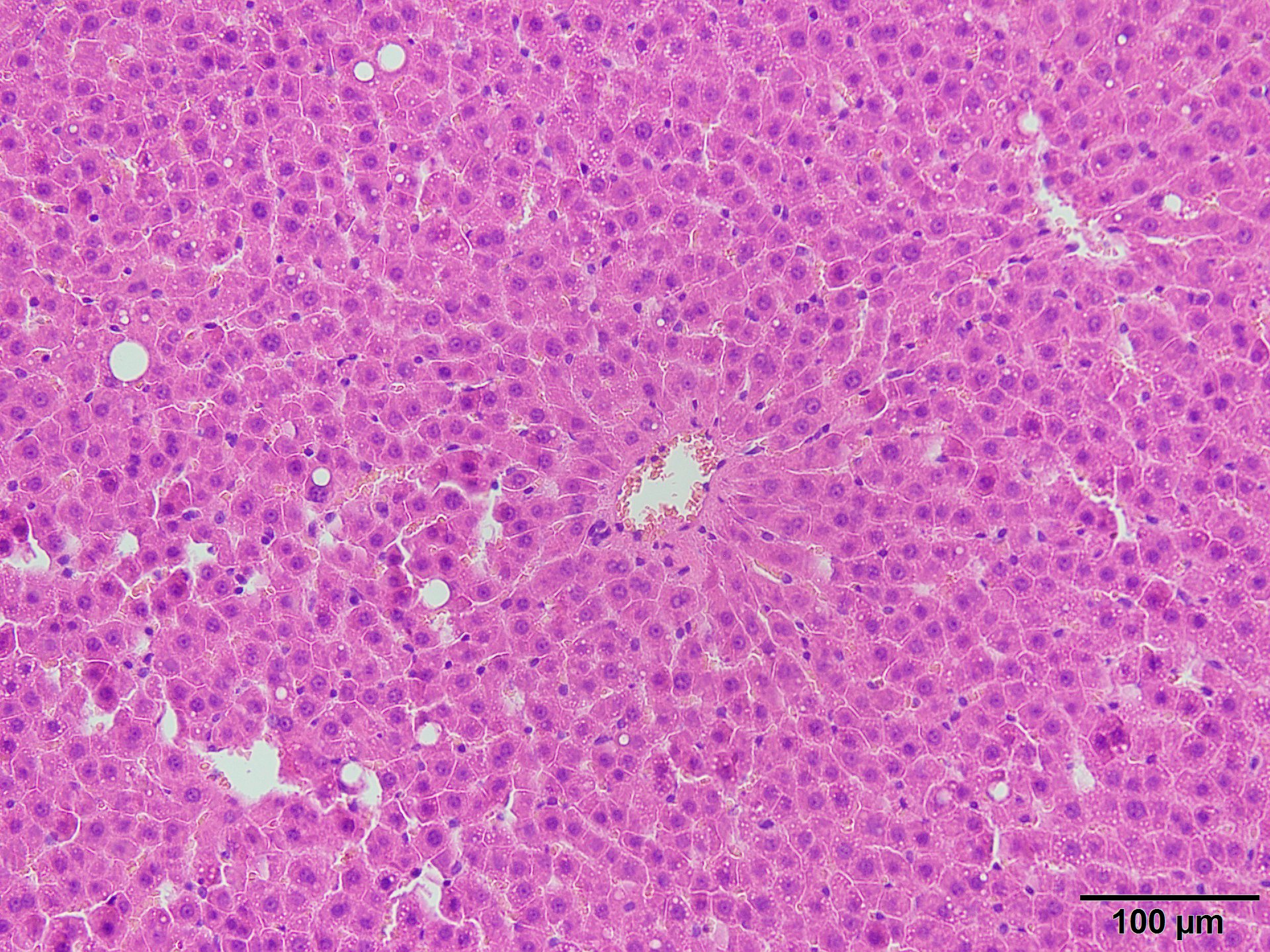

Supplement: Supplementary file 1 [file Data_Sheet_1.ZIP › SCHY34-raw data/figure 5/EDTA.jpg]

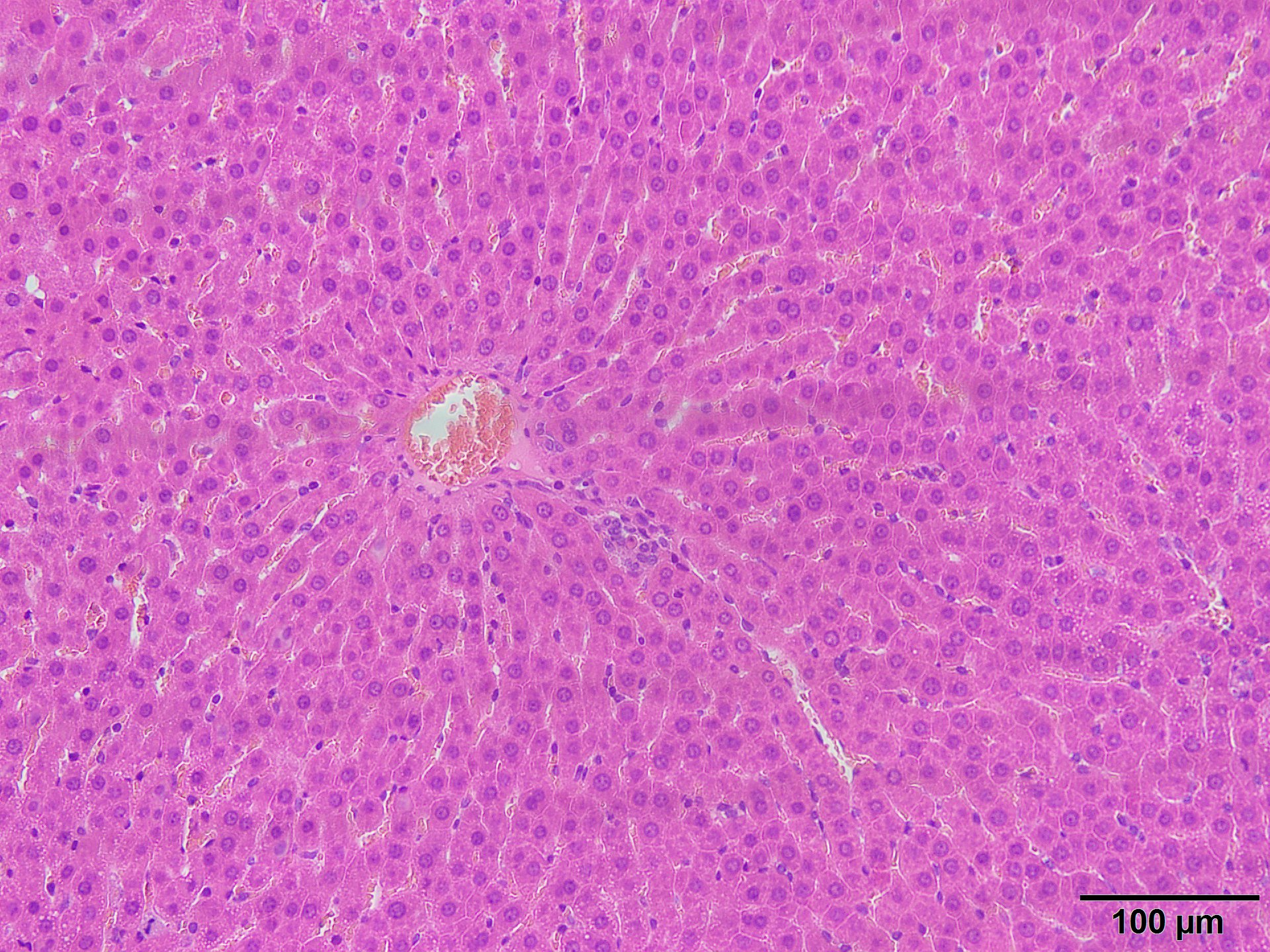

Supplement: Supplementary file 1 [file Data_Sheet_1.ZIP › SCHY34-raw data/figure 5/LF-SCHY34.jpg]

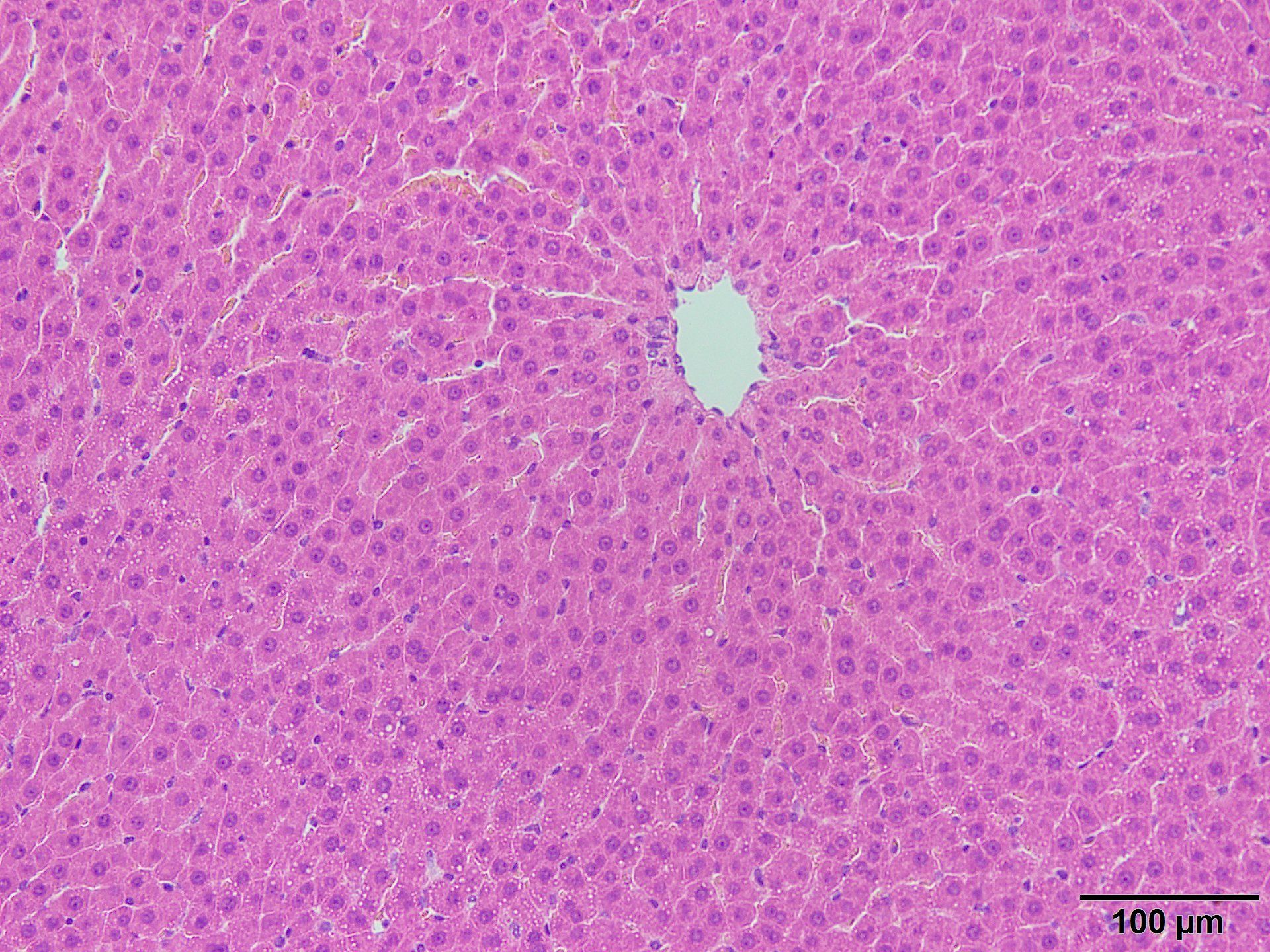

Supplement: Supplementary file 1 [file Data_Sheet_1.ZIP › SCHY34-raw data/figure 5/Normal.jpg]

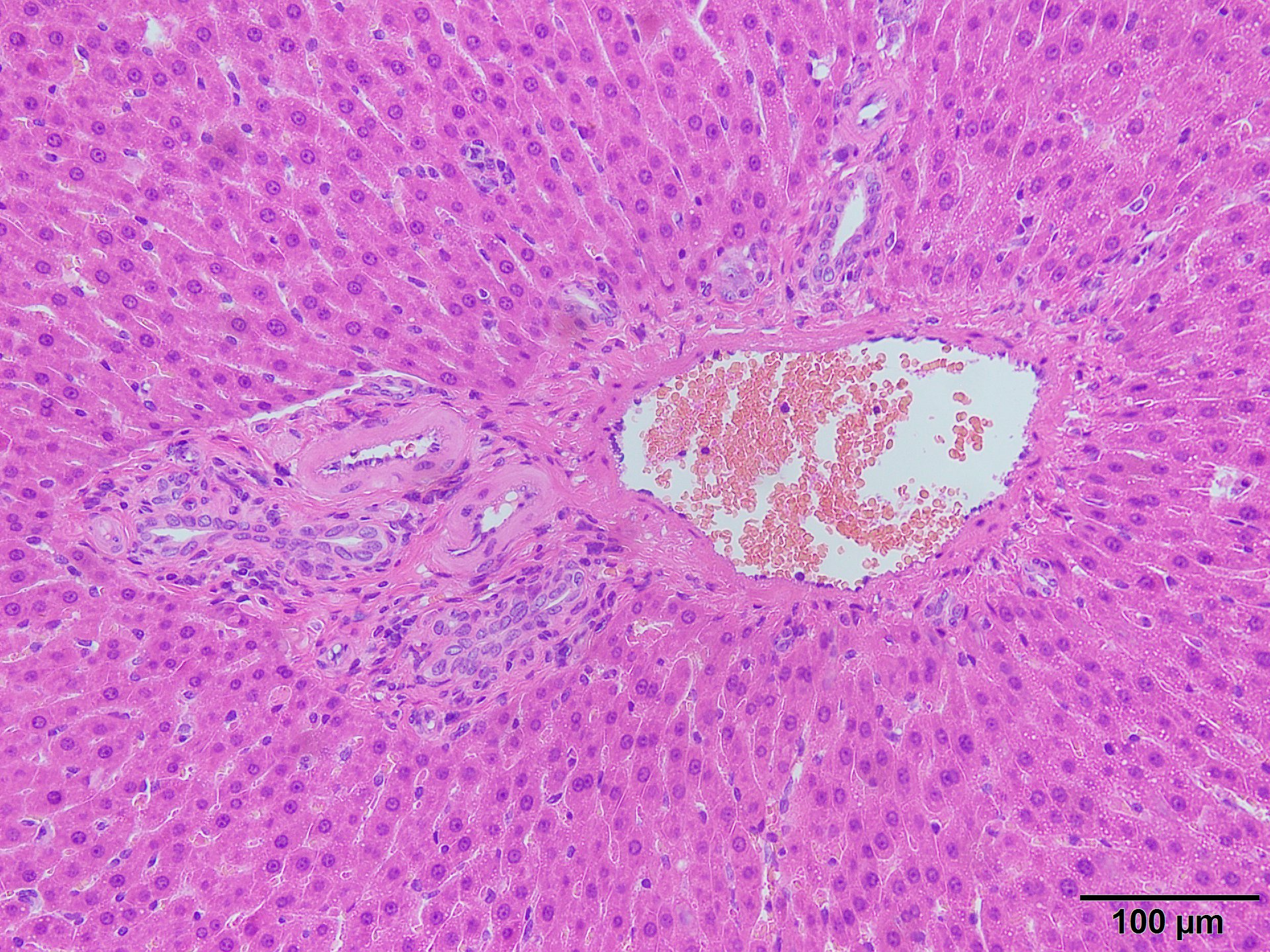

Supplement: Supplementary file 1 [file Data_Sheet_1.ZIP › SCHY34-raw data/figure 5/lead-induced.jpg]

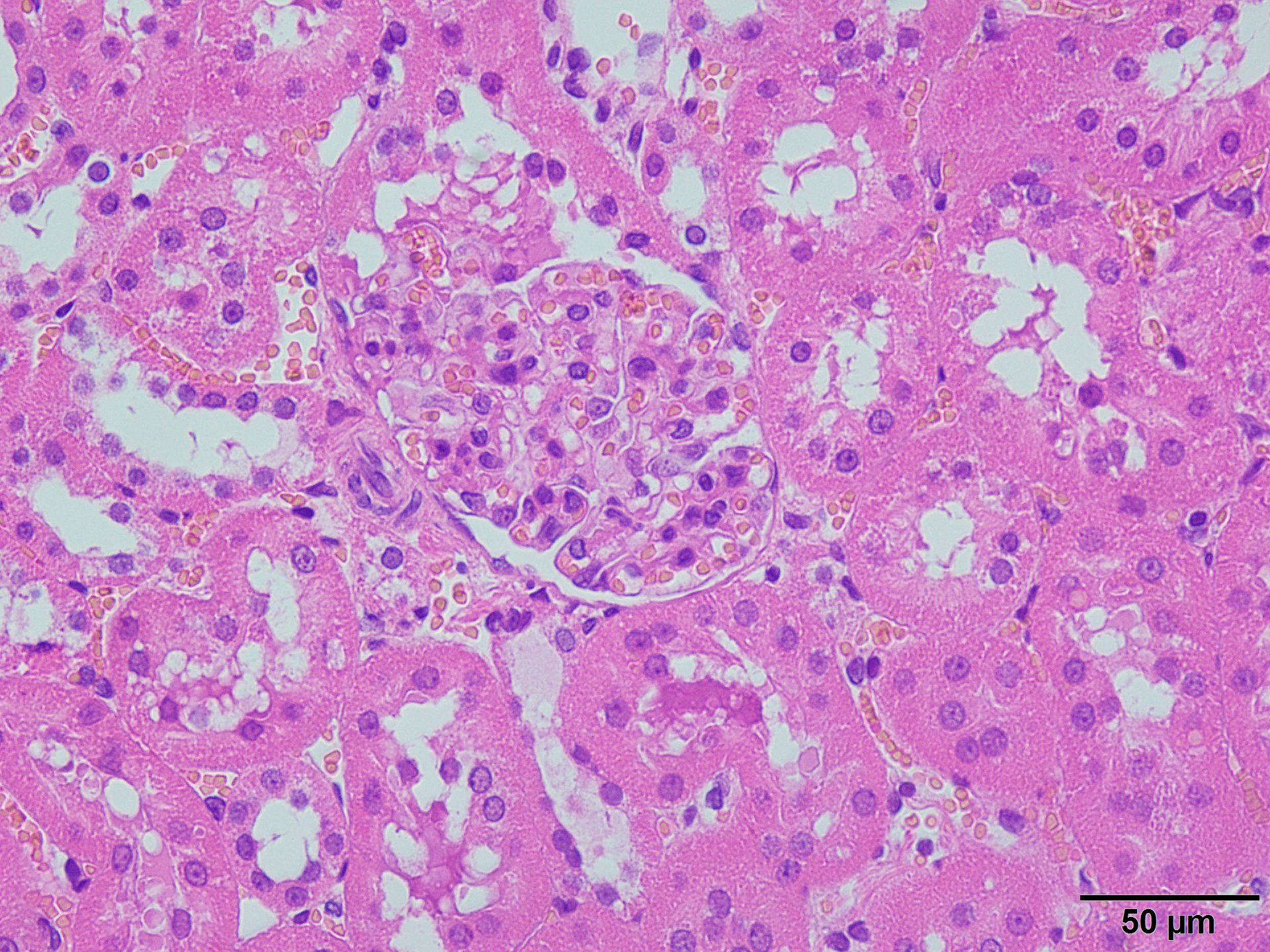

Supplement: Supplementary file 1 [file Data_Sheet_1.ZIP › SCHY34-raw data/figure 6/EDTA.jpg]

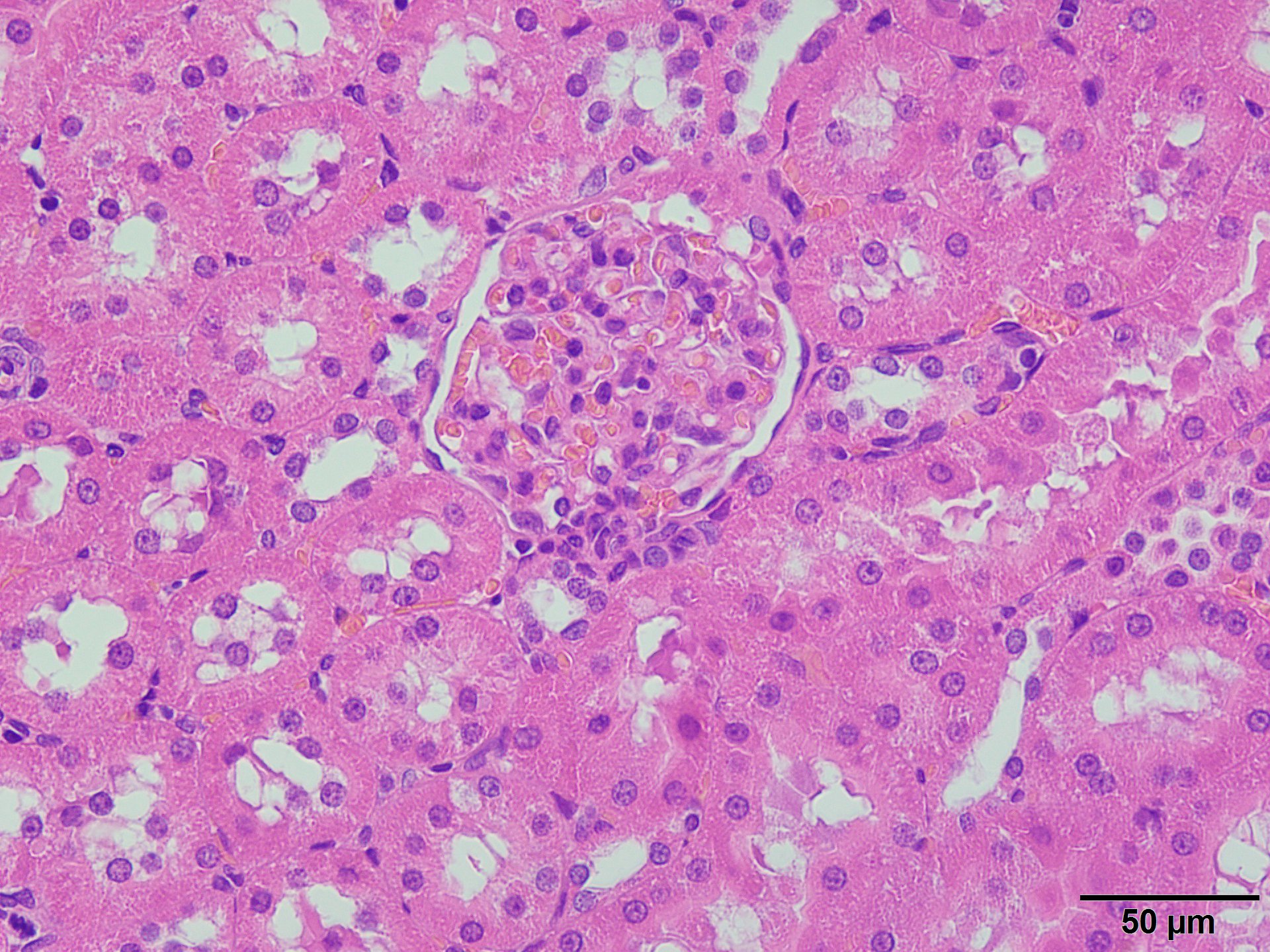

Supplement: Supplementary file 1 [file Data_Sheet_1.ZIP › SCHY34-raw data/figure 6/LF-SCHY34.jpg]

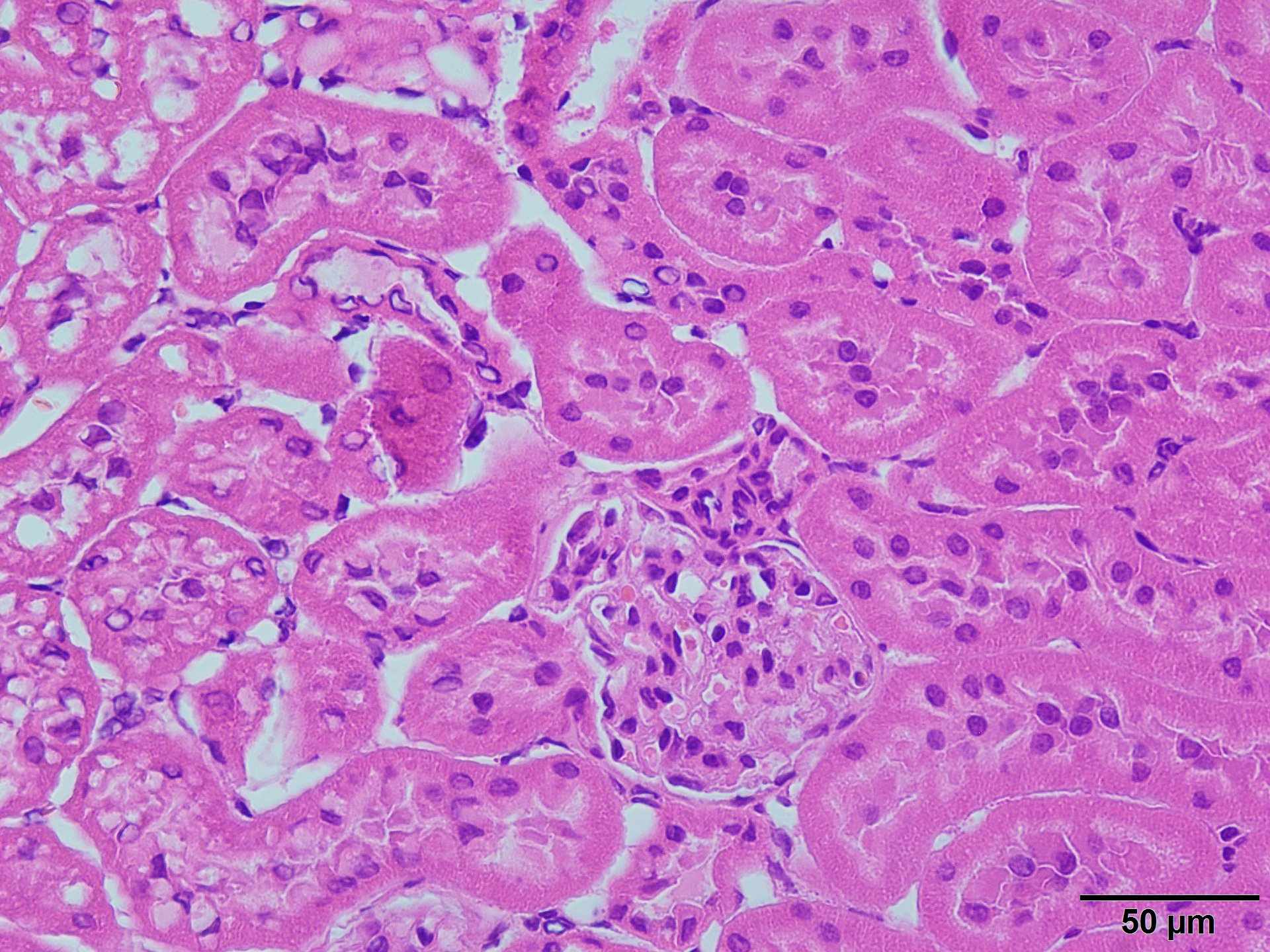

Supplement: Supplementary file 1 [file Data_Sheet_1.ZIP › SCHY34-raw data/figure 6/Lead-induced.jpg]

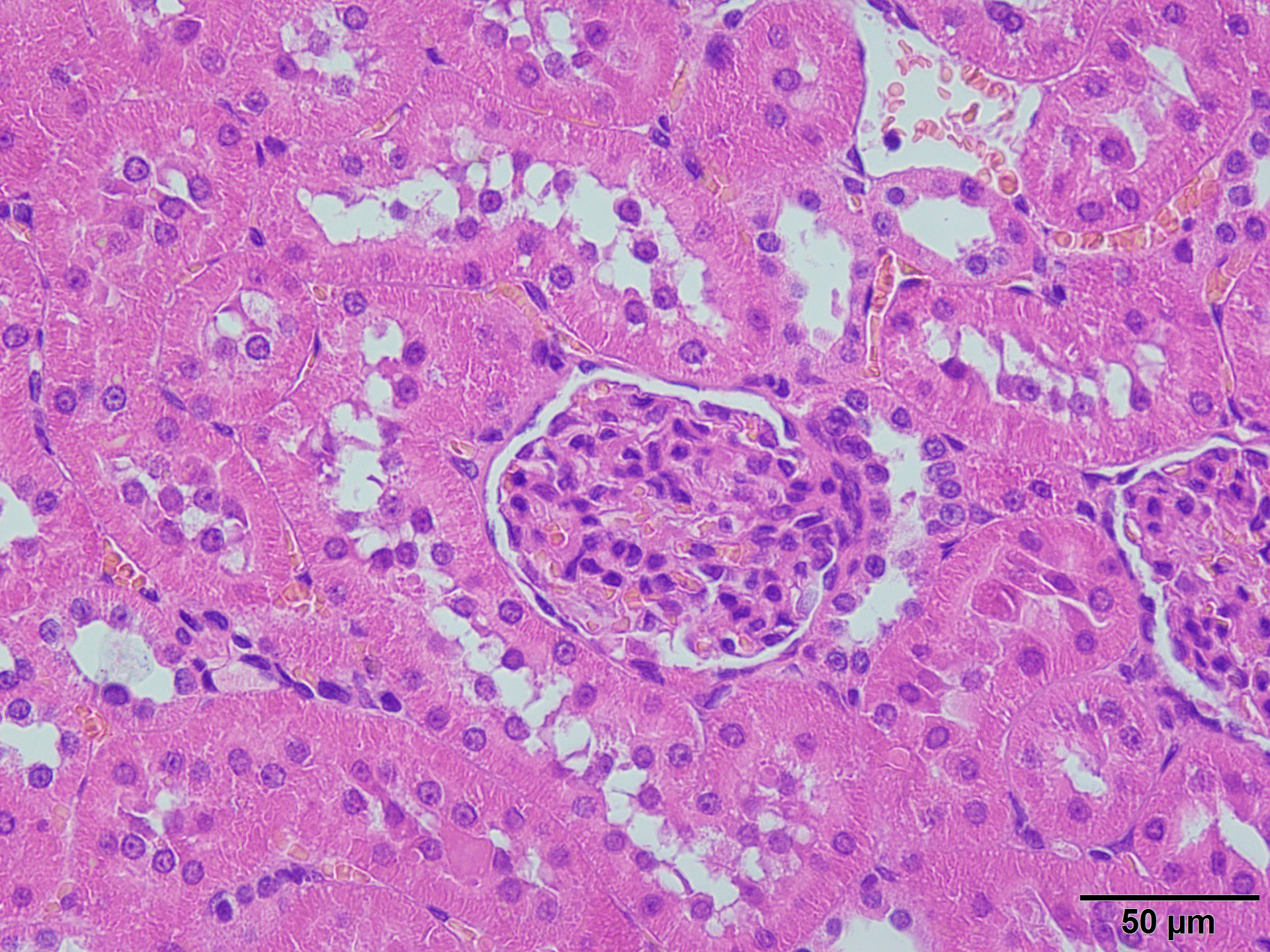

Supplement: Supplementary file 1 [file Data_Sheet_1.ZIP › SCHY34-raw data/figure 6/Normal.jpg]

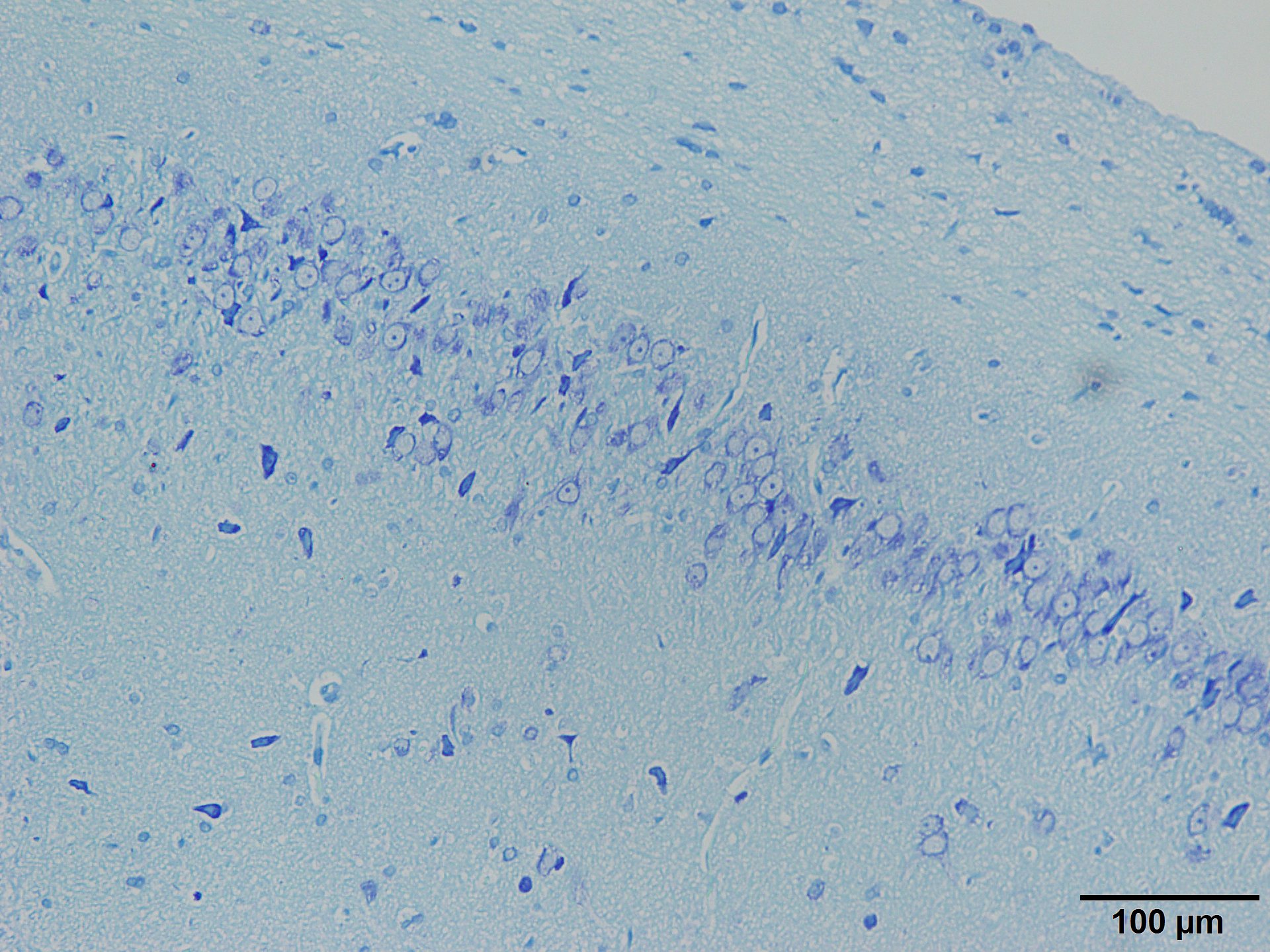

Supplement: Supplementary file 1 [file Data_Sheet_1.ZIP › SCHY34-raw data/figure 7/CA1-EDTA║ú┬φ15-8.jpg]

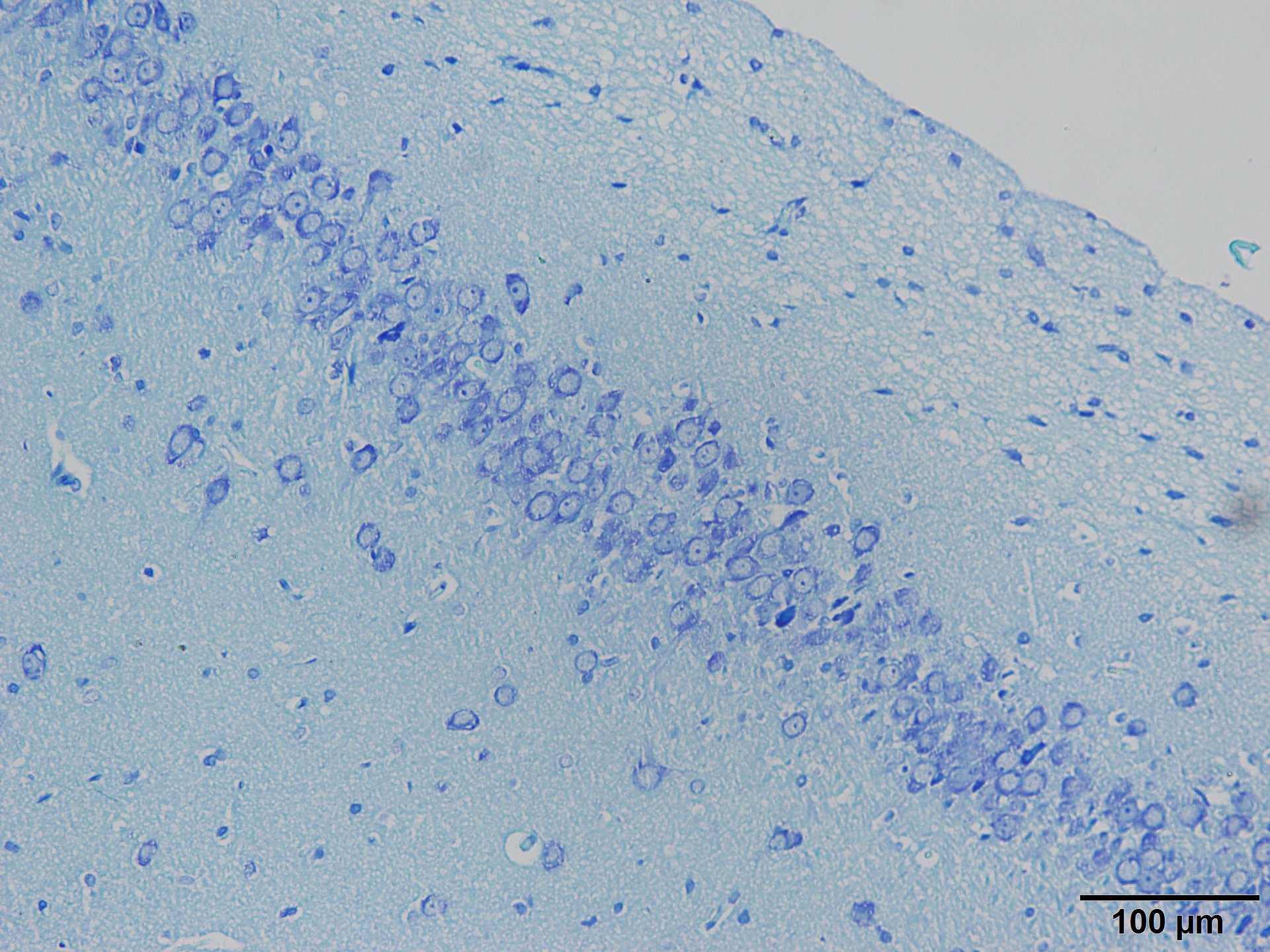

Supplement: Supplementary file 1 [file Data_Sheet_1.ZIP › SCHY34-raw data/figure 7/CA1-LF-SCHY34║ú┬φ6-6.jpg]

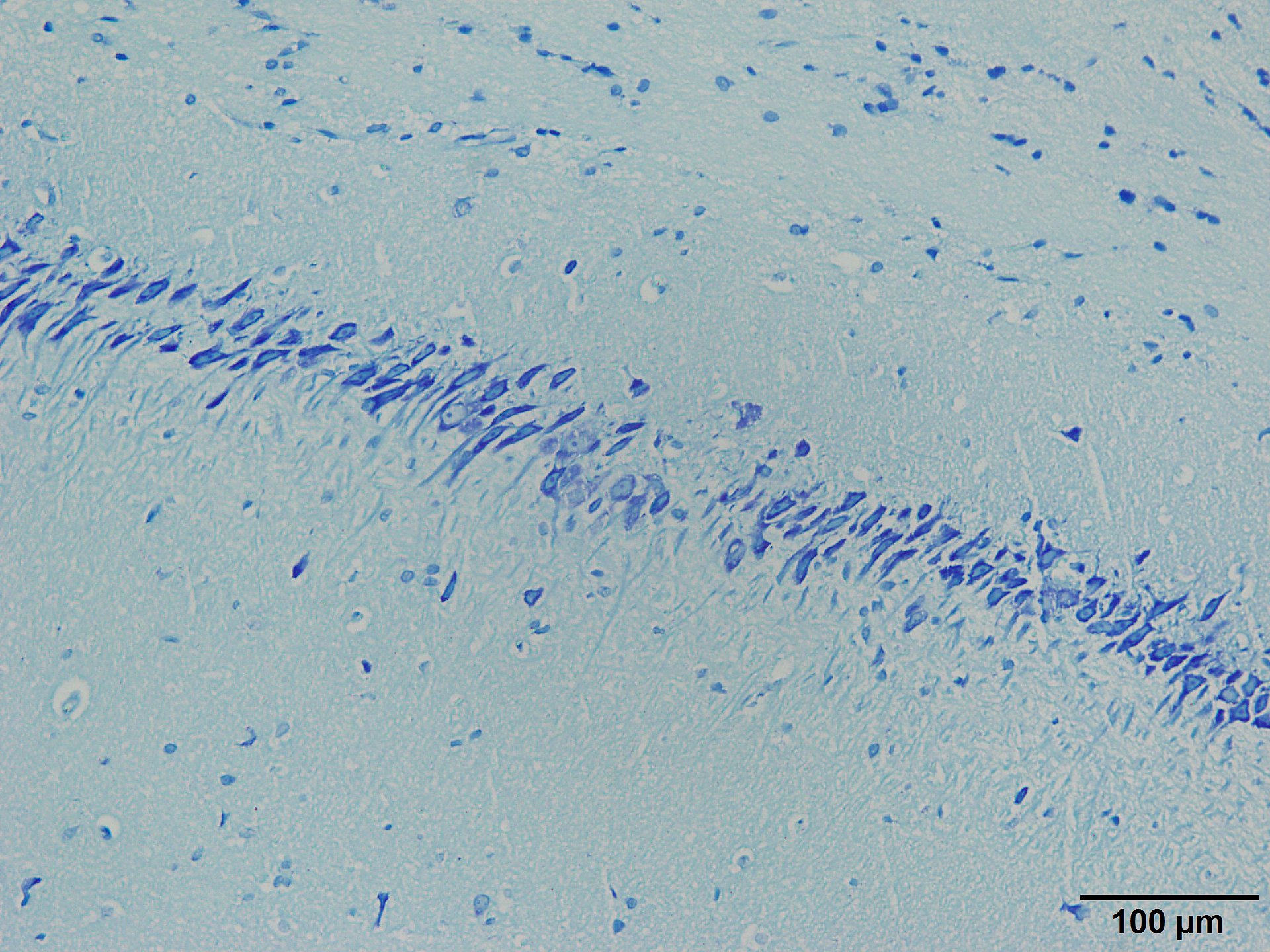

Supplement: Supplementary file 1 [file Data_Sheet_1.ZIP › SCHY34-raw data/figure 7/CA1-Lead-induced║ú┬φ12-3.jpg]

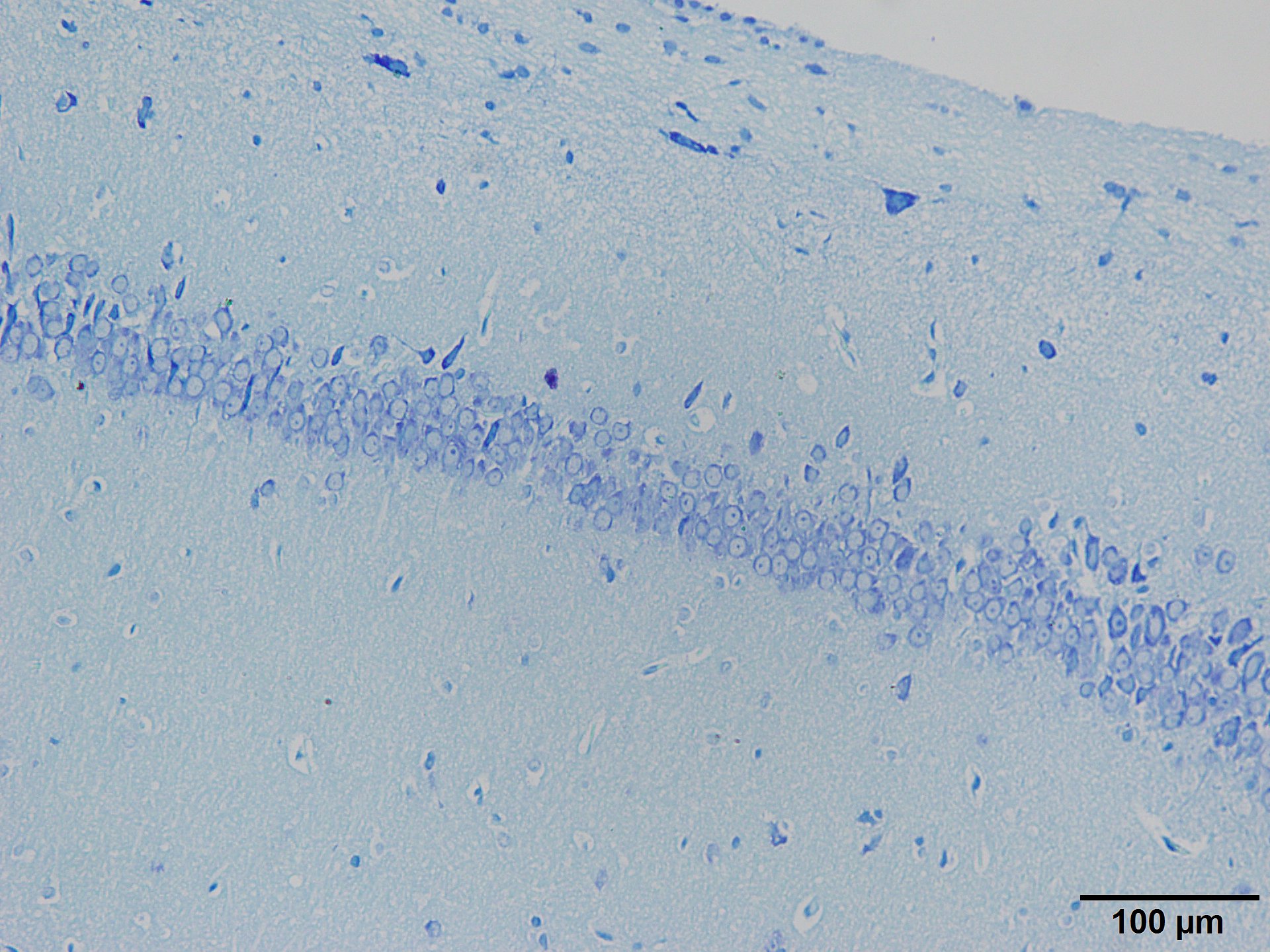

Supplement: Supplementary file 1 [file Data_Sheet_1.ZIP › SCHY34-raw data/figure 7/CA1-Normal║ú┬φ1-5.jpg]

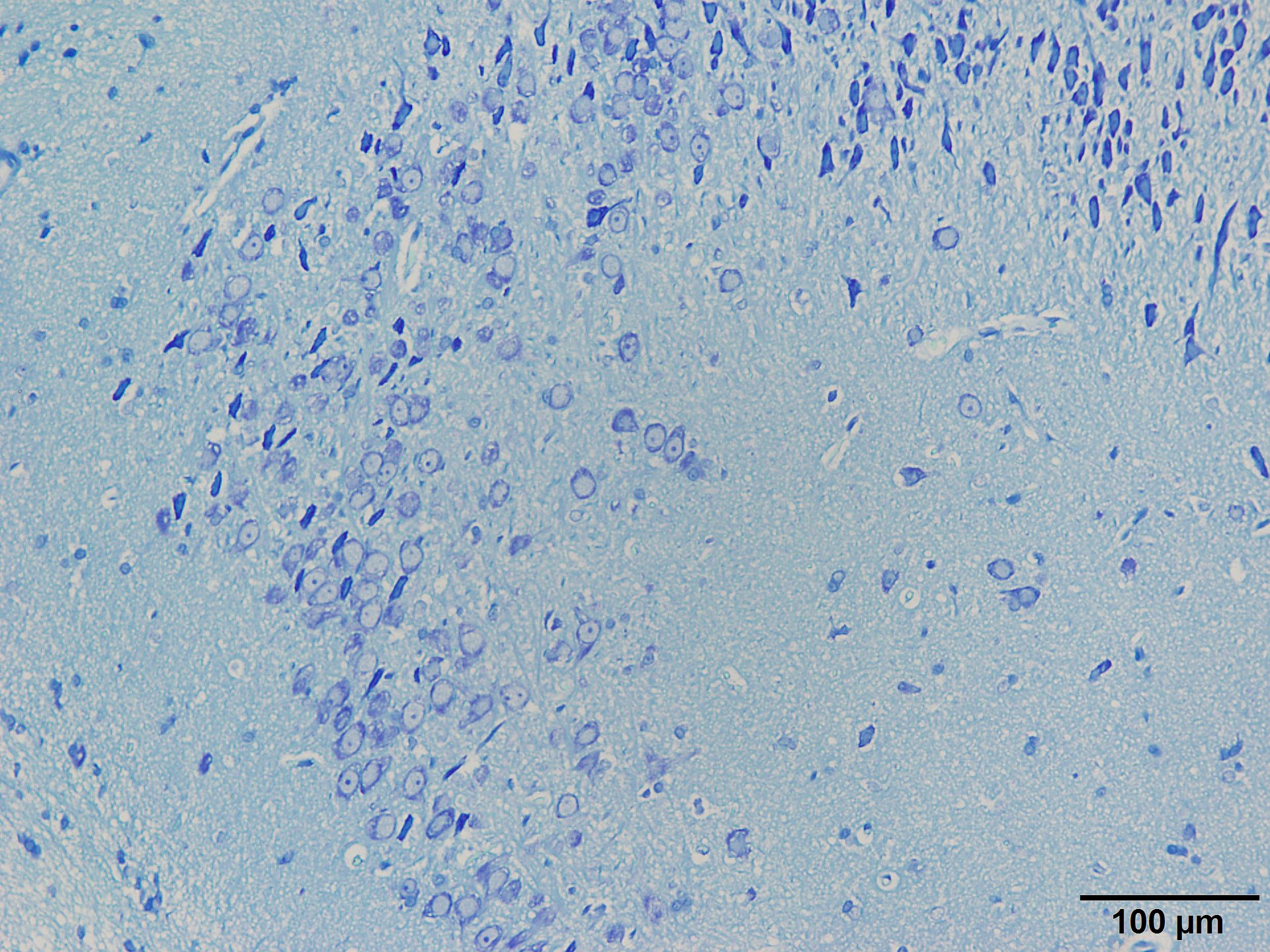

Supplement: Supplementary file 1 [file Data_Sheet_1.ZIP › SCHY34-raw data/figure 7/CA3-EDTA.jpg]

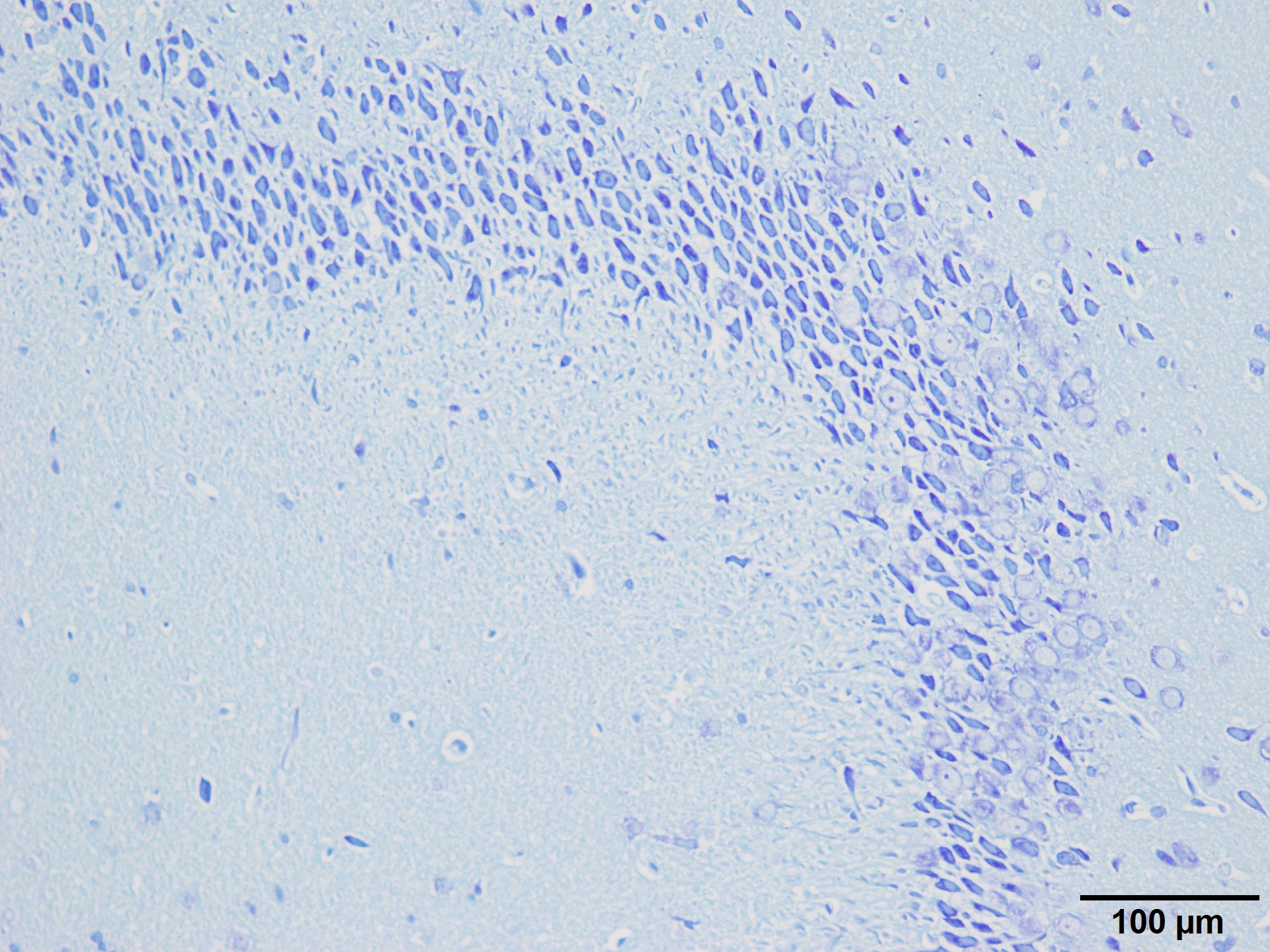

Supplement: Supplementary file 1 [file Data_Sheet_1.ZIP › SCHY34-raw data/figure 7/CA3-LF-SCHY34.jpg]

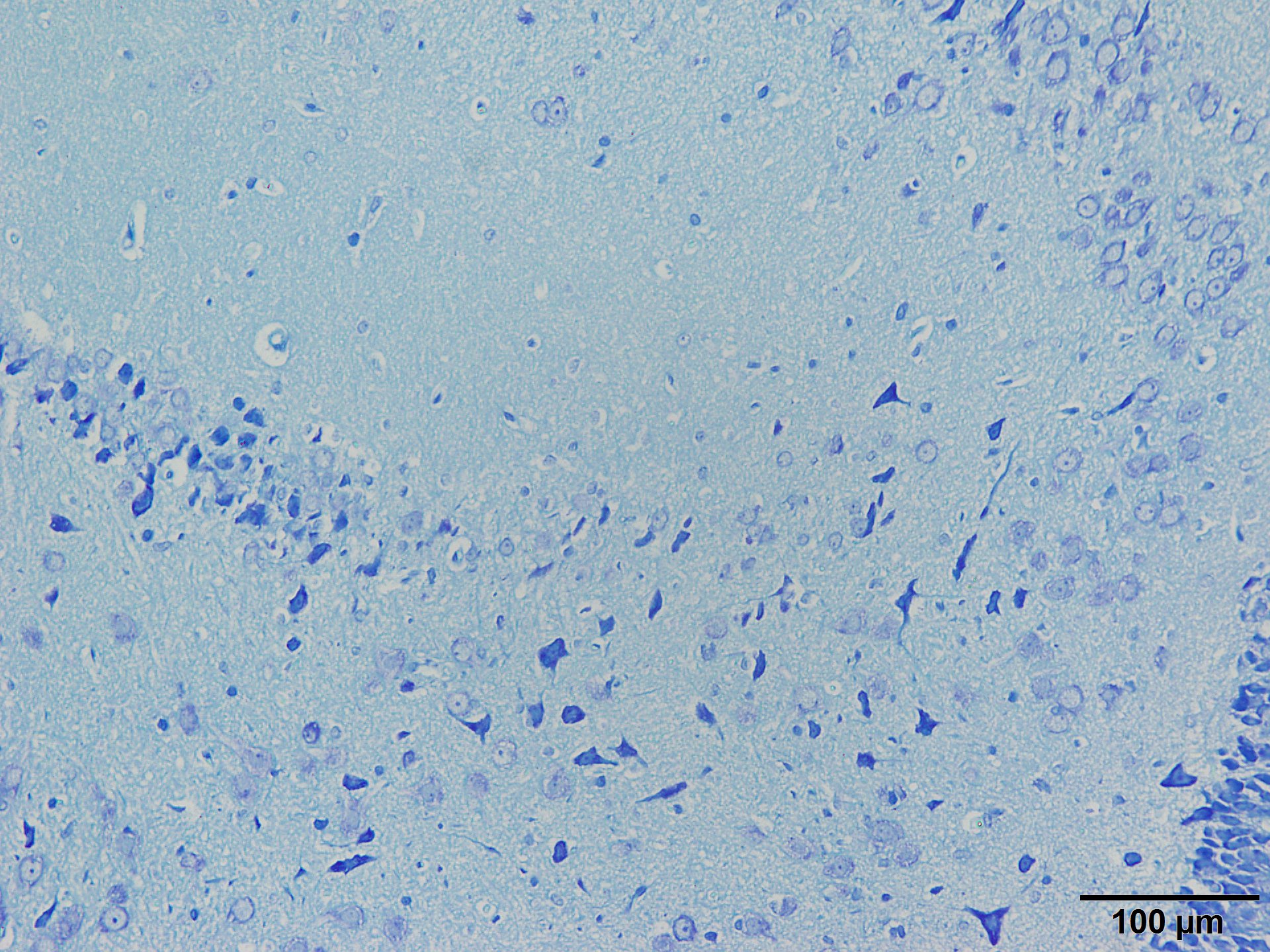

Supplement: Supplementary file 1 [file Data_Sheet_1.ZIP › SCHY34-raw data/figure 7/CA3-Lead-induced.jpg]

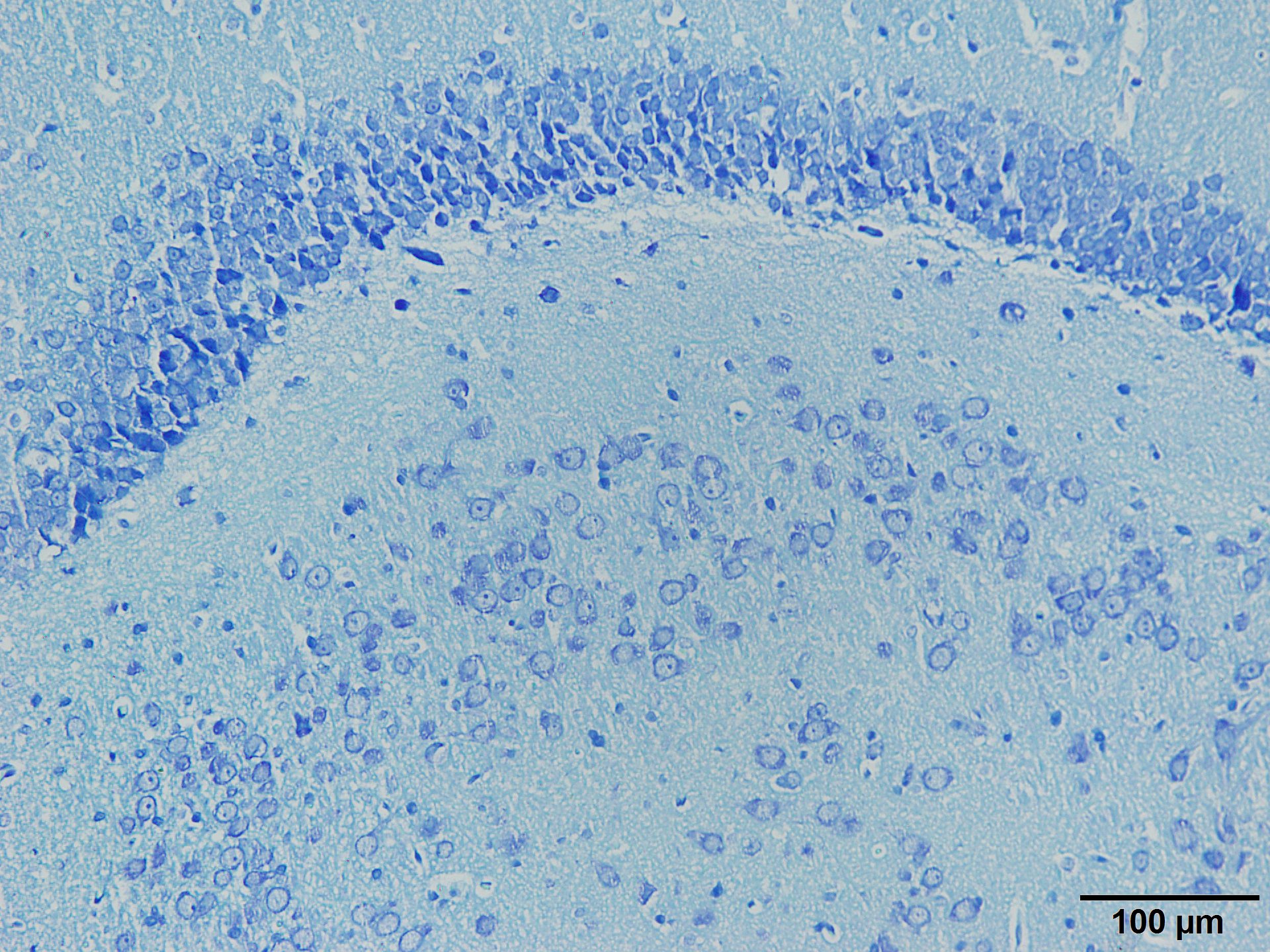

Supplement: Supplementary file 1 [file Data_Sheet_1.ZIP › SCHY34-raw data/figure 7/CA3-Normal.jpg]

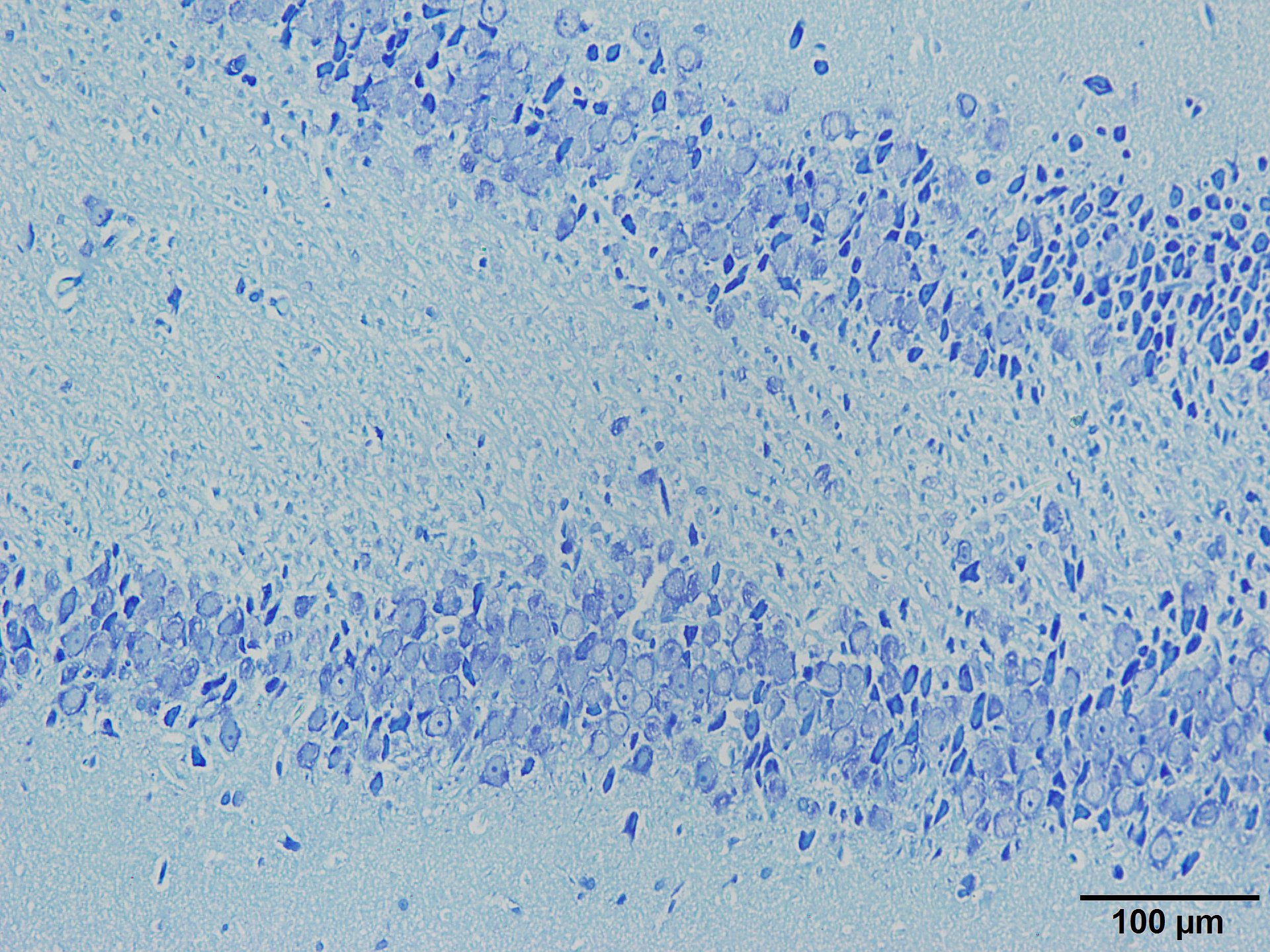

Supplement: Supplementary file 1 [file Data_Sheet_1.ZIP › SCHY34-raw data/figure 7/DG-EDTA.jpg]

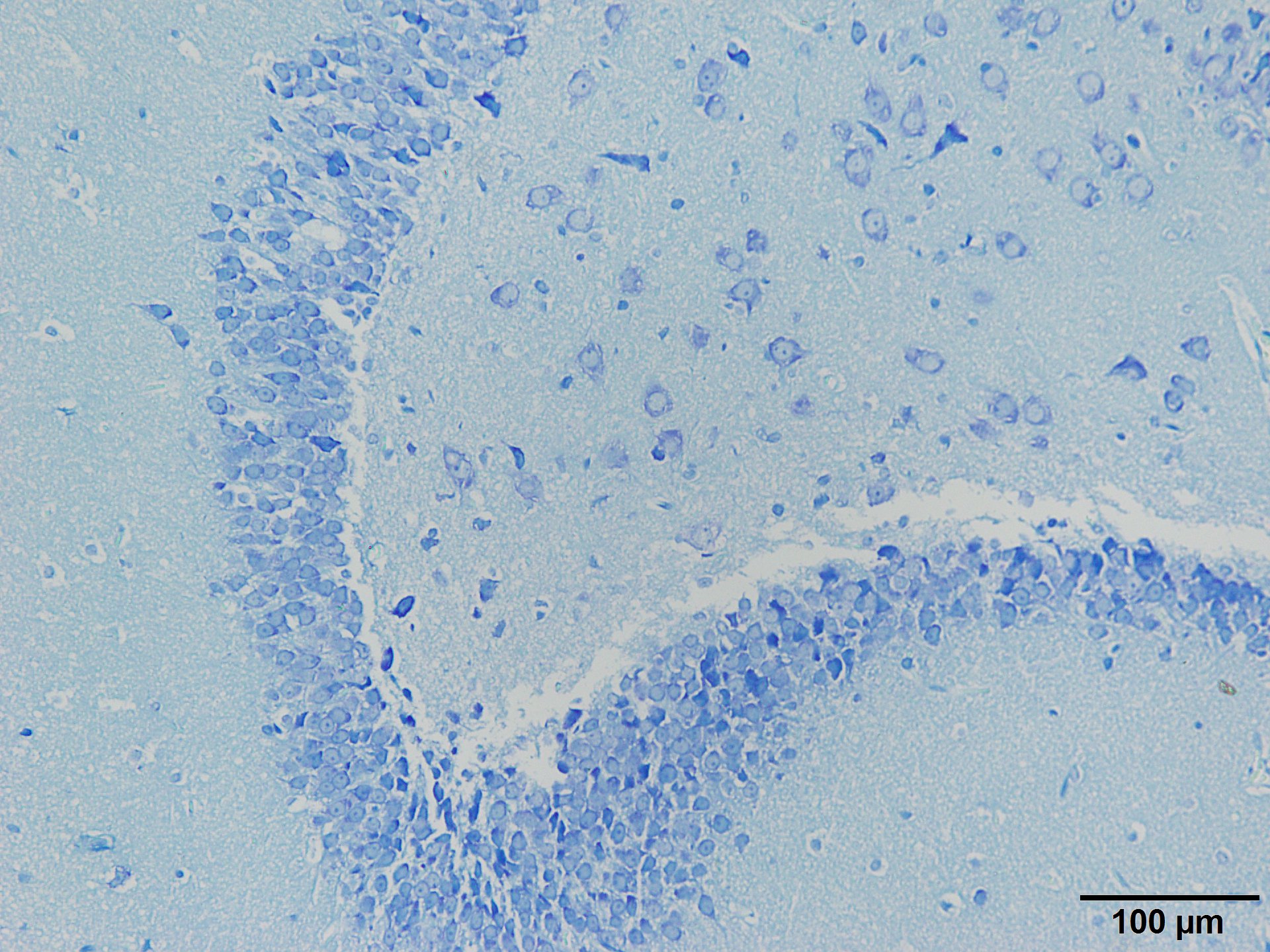

Supplement: Supplementary file 1 [file Data_Sheet_1.ZIP › SCHY34-raw data/figure 7/DG-LF-SCHY34.jpg]

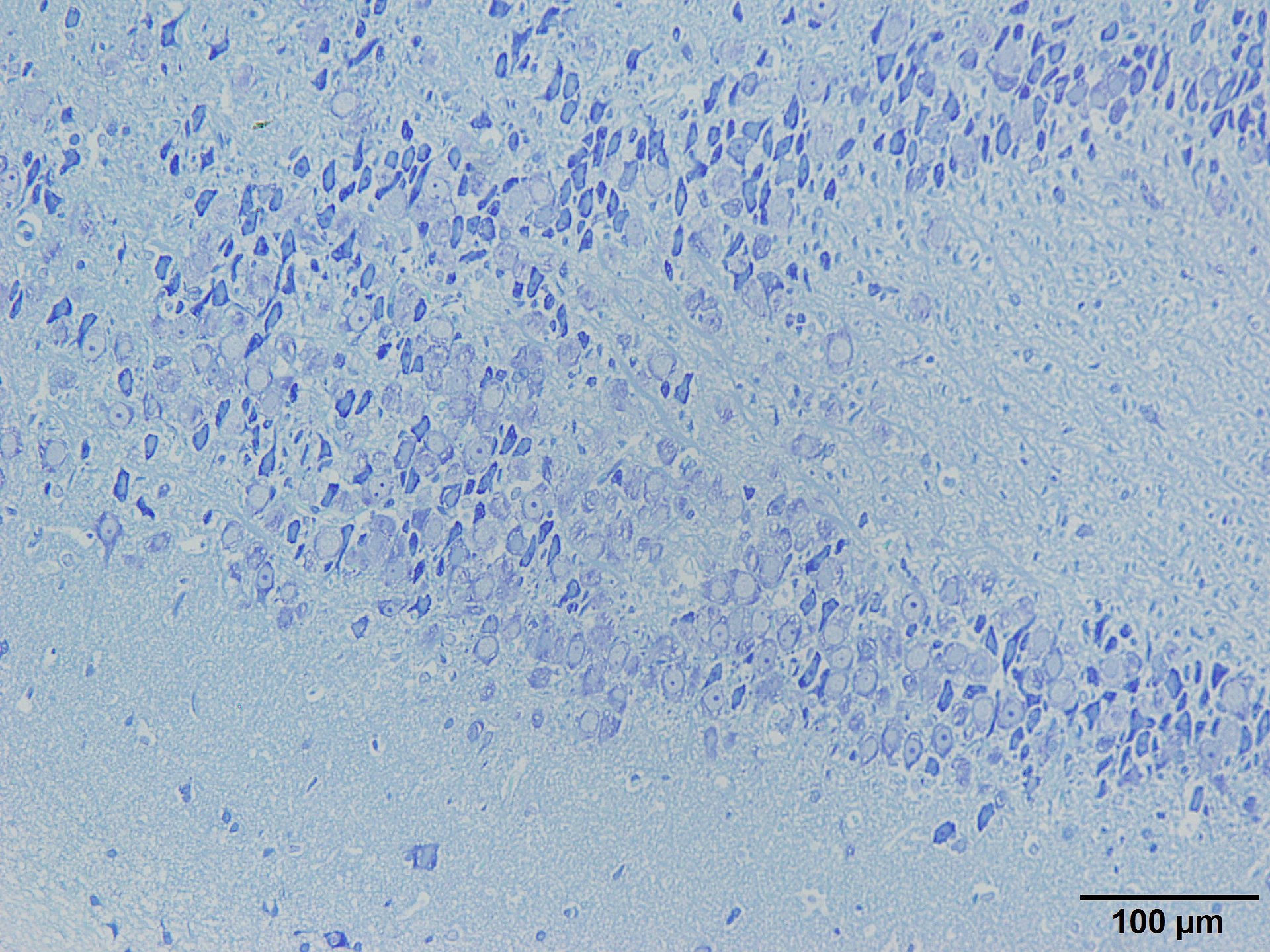

Supplement: Supplementary file 1 [file Data_Sheet_1.ZIP › SCHY34-raw data/figure 7/DG-Lead-induced.jpg]

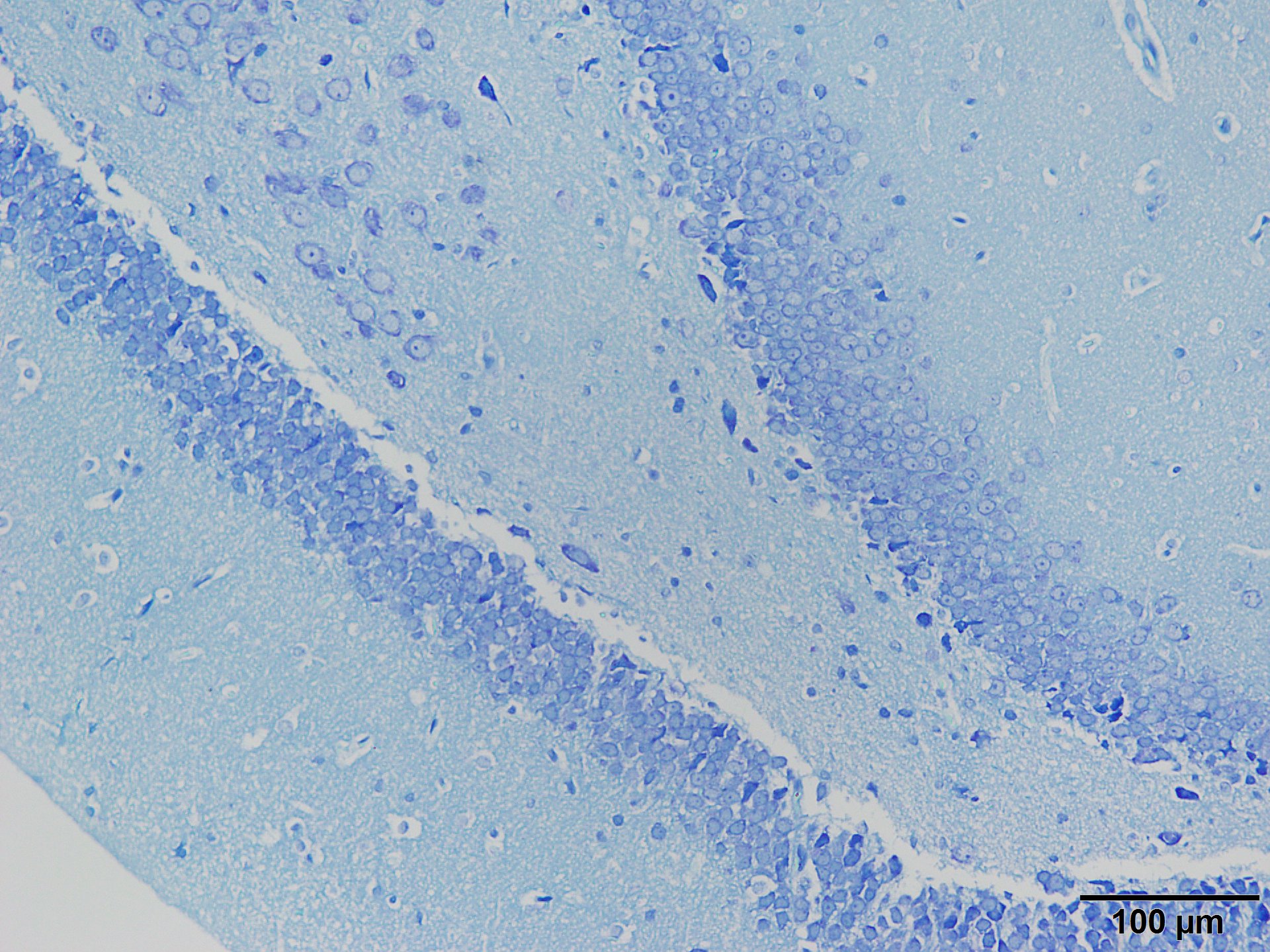

Supplement: Supplementary file 1 [file Data_Sheet_1.ZIP › SCHY34-raw data/figure 7/DG-Normal.jpg]

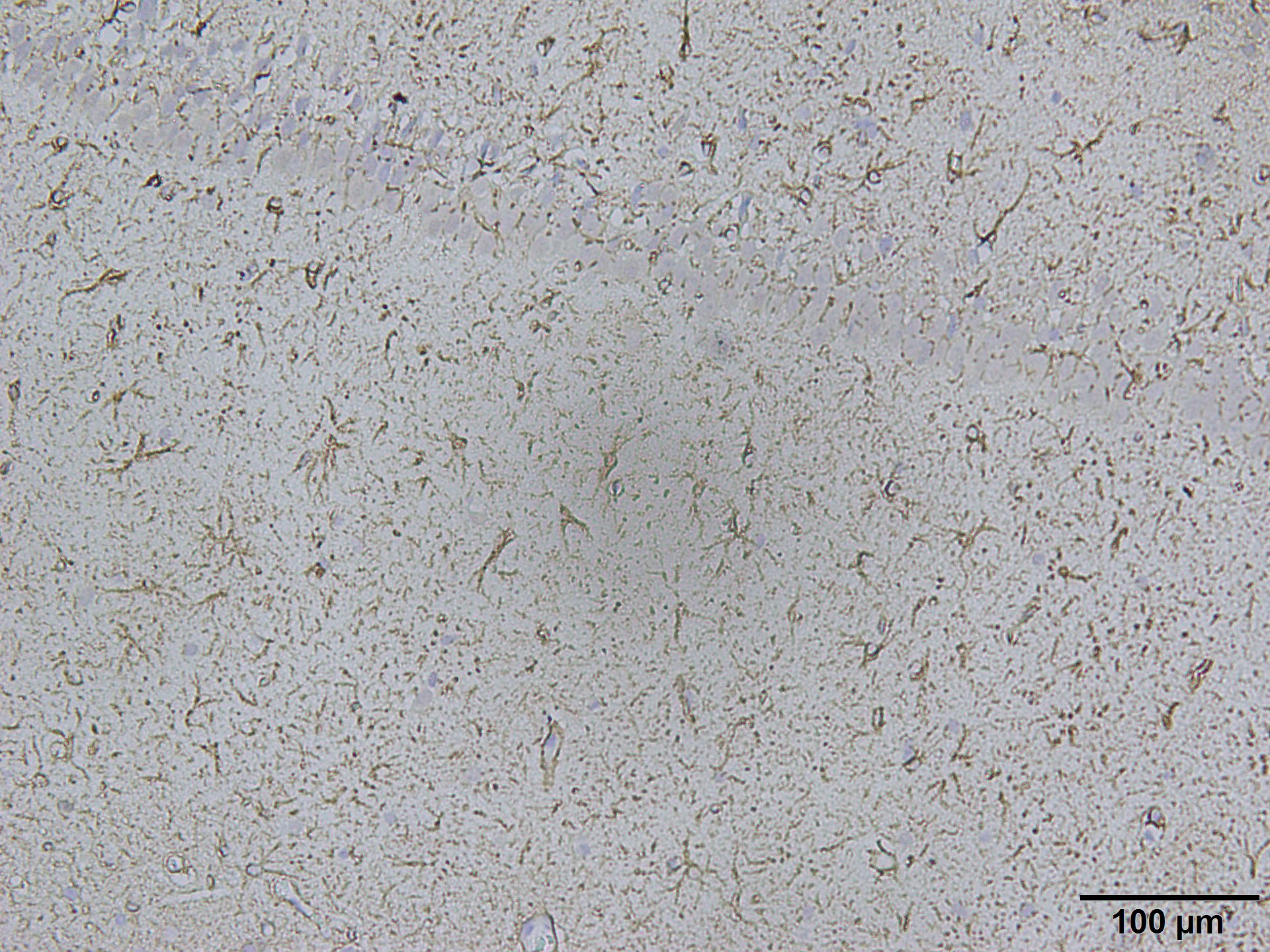

Supplement: Supplementary file 1 [file Data_Sheet_1.ZIP › SCHY34-raw data/figure 8/EDTA.jpg]

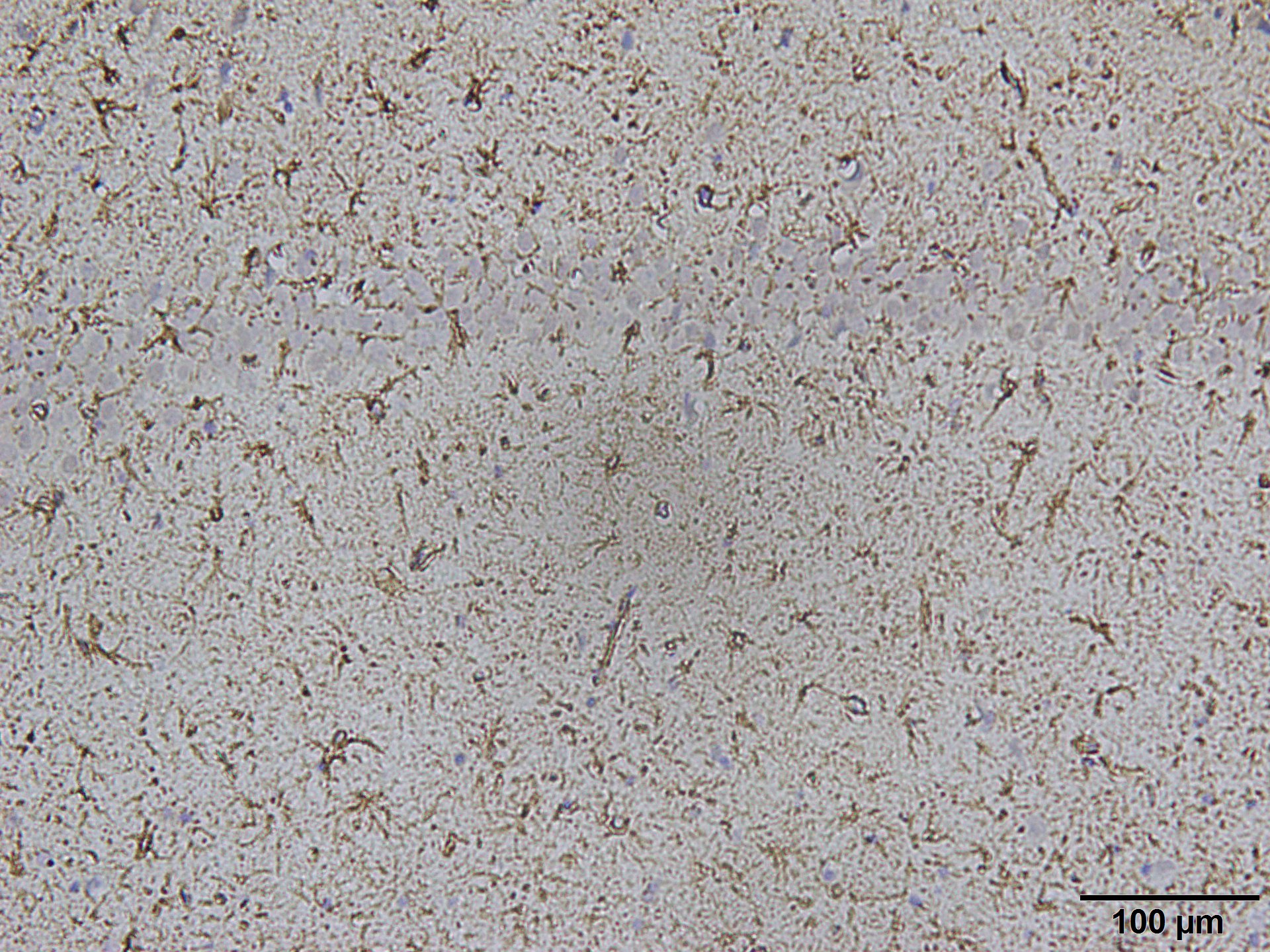

Supplement: Supplementary file 1 [file Data_Sheet_1.ZIP › SCHY34-raw data/figure 8/LF-SCHY34.jpg]

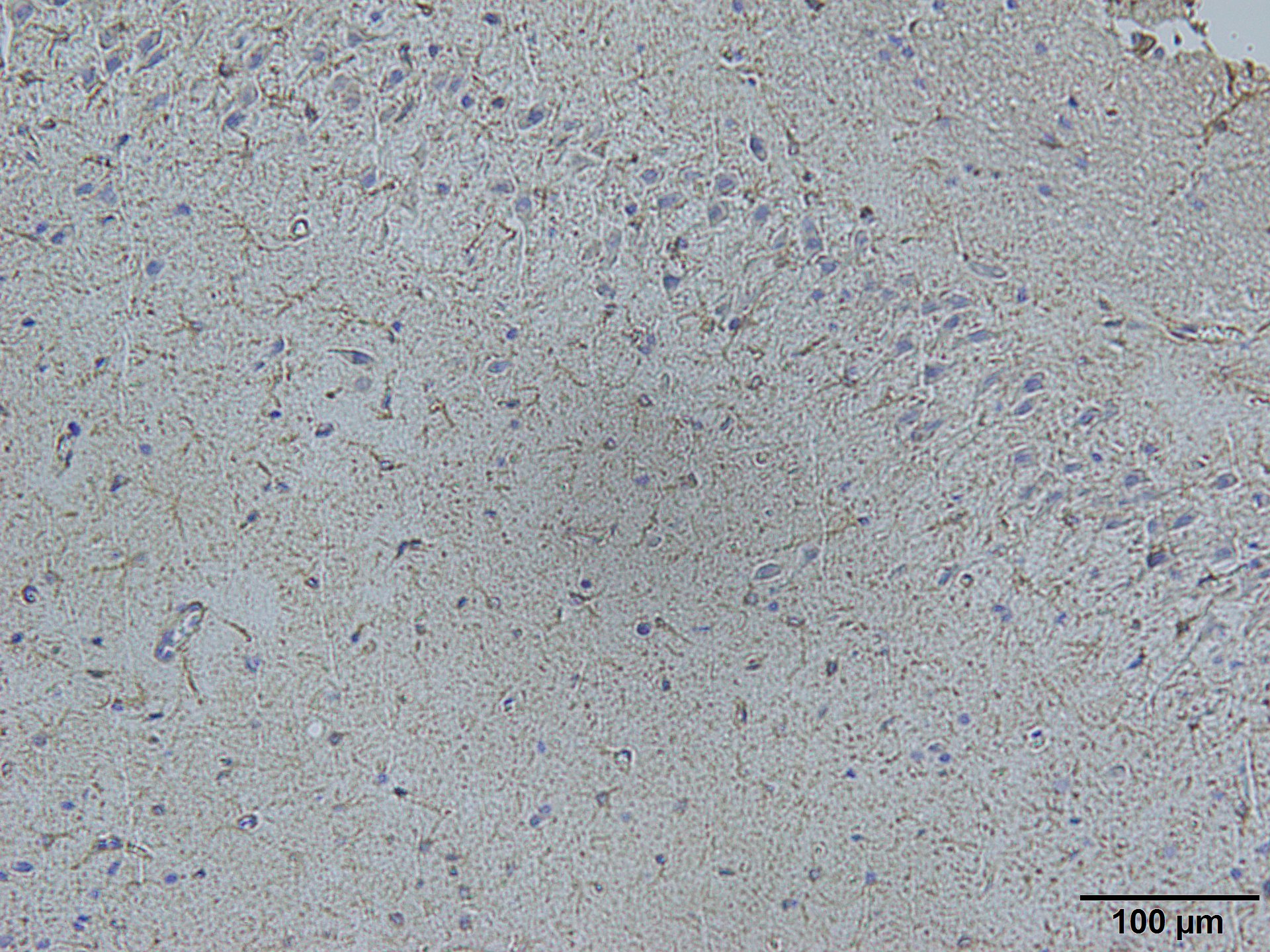

Supplement: Supplementary file 1 [file Data_Sheet_1.ZIP › SCHY34-raw data/figure 8/Lead-induced.jpg]

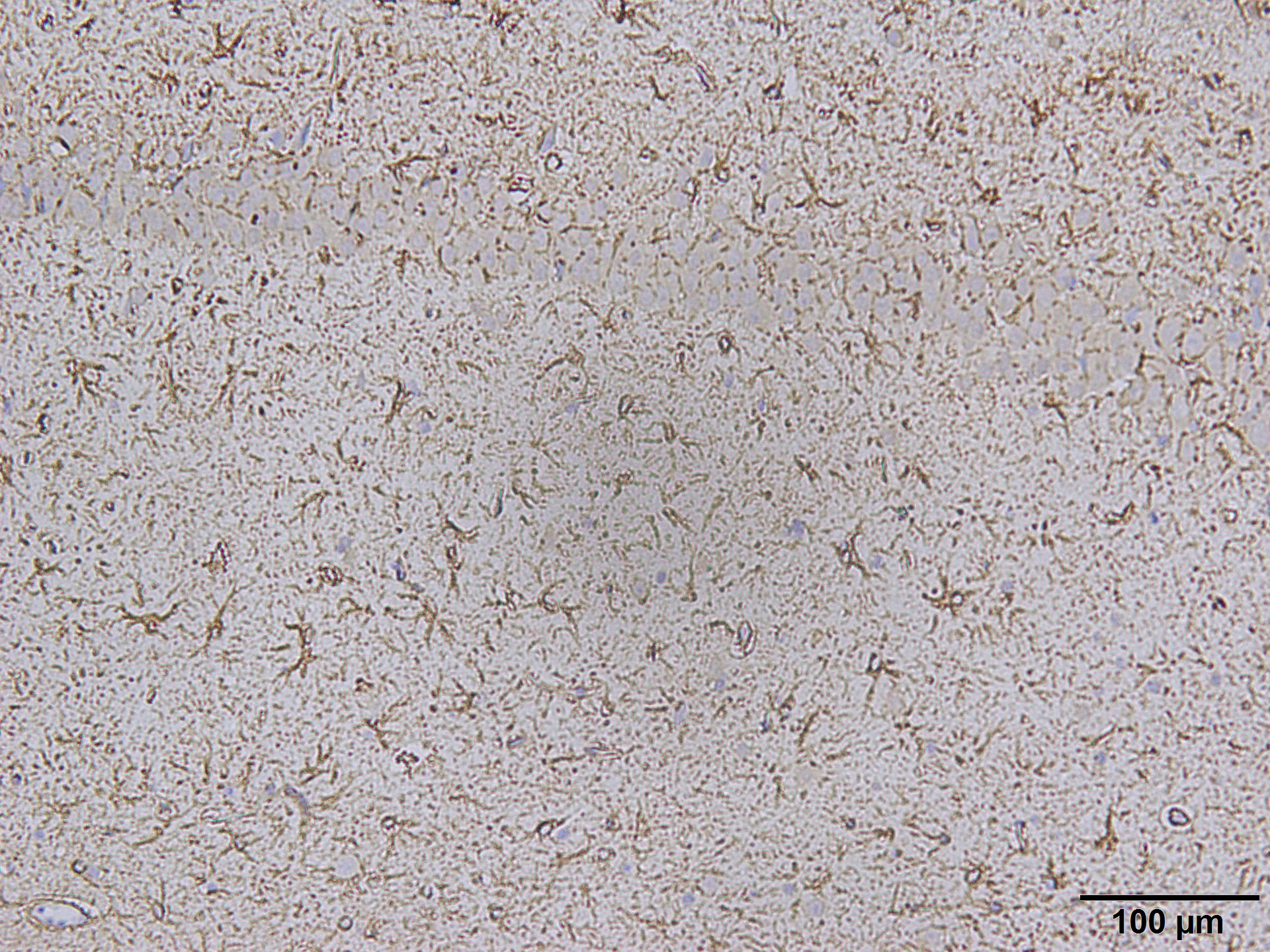

Supplement: Supplementary file 1 [file Data_Sheet_1.ZIP › SCHY34-raw data/figure 8/Normal.jpg]

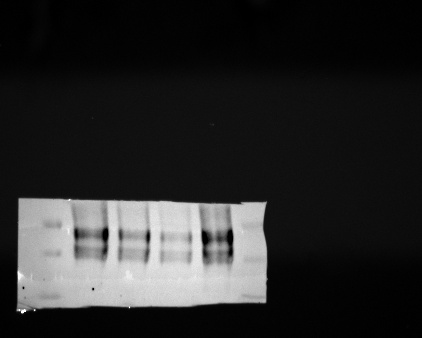

Supplement: Supplementary file 1 [file Data_Sheet_1.ZIP › SCHY34-raw data/figure 9/BDNF.jpg]

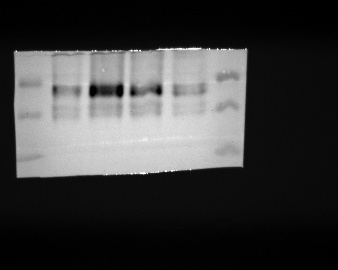

Supplement: Supplementary file 1 [file Data_Sheet_1.ZIP › SCHY34-raw data/figure 9/Bax.jpg]

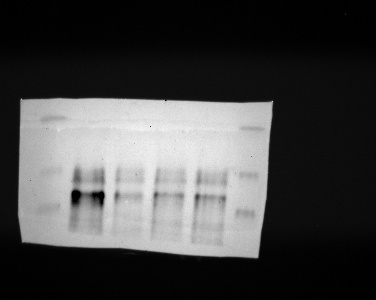

Supplement: Supplementary file 1 [file Data_Sheet_1.ZIP › SCHY34-raw data/figure 9/Bcl-2.jpg]

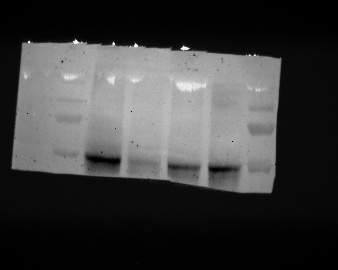

Supplement: Supplementary file 1 [file Data_Sheet_1.ZIP › SCHY34-raw data/figure 9/CAM.jpg]

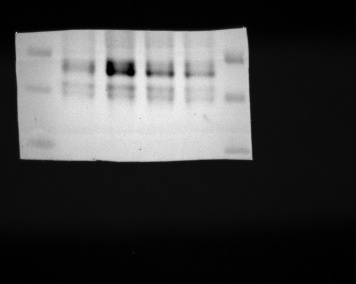

Supplement: Supplementary file 1 [file Data_Sheet_1.ZIP › SCHY34-raw data/figure 9/Caspase-3.jpg]

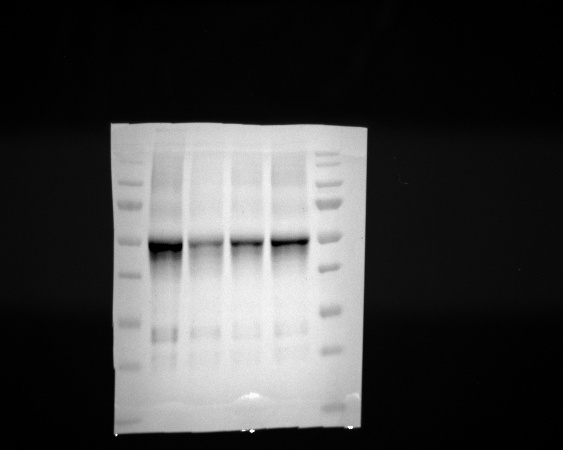

Supplement: Supplementary file 1 [file Data_Sheet_1.ZIP › SCHY34-raw data/figure 9/GSH.jpg]

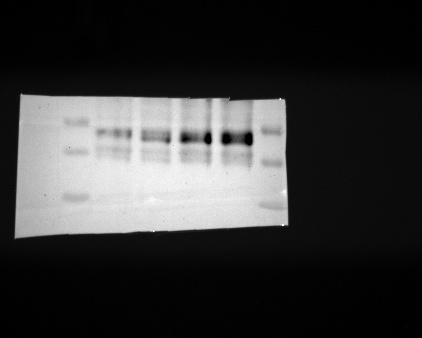

Supplement: Supplementary file 1 [file Data_Sheet_1.ZIP › SCHY34-raw data/figure 9/HO_1.jpg]
